# UPLC‐Q‐TOF‐MS/MS Profiling and Synergistic Anti‐HCC Activity of Gallic Acid From Compound Muji Granules Combined With hUC‐MSC‐Exos

**DOI:** 10.1002/cbdv.71809

**Published:** 2026-10-11

**Authors:** Qiang Zhang, Yige Wang, Jinnan Huo, Liying Han, Ying Meng, Yuanyuan Li, Jiaxin Shi, Xi Wu, Yongrui Bao, Shuai Wang, Tianjiao Li, Jiapeng Leng, Xiansheng Meng

**Affiliations:** ^1^ College of Pharmacy Liaoning University of Traditional Chinese Medicine Dalian Liaoning China; ^2^ College of Acupuncture‐Moxibustion and Tuina Liaoning University of Traditional Chinese Medicine Shenyang Liaoning China; ^3^ Professional Innovation Technology Center for Multidimensional Analysis of Traditional Chinese Medicine Dalian Liaoning China; ^4^ Liaoning Province Modern Chinese Medicine Research Engineering Laboratory Dalian Liaoning China

**Keywords:** chemical constituents, Compound Muji Granules, exosomes, gallic acid, hepatocellular carcinoma

## Abstract

Hepatocellular carcinoma (HCC) lacks effective therapies. Compound Muji Granules (MJ), a traditional Chinese medicine, shows potential anti‐HCC activity, but its material basis and synergistic mechanism with exosomal delivery remain unclear. Using UPLC‐Q‐TOF‐MS/MS, 98 compounds were identified in MJ, including 25 blood‐absorbed and 23 tissue‐distributed components, with gallic acid (GA) as the core active constituent. hUC‐MSC‐derived exosomes (hUC‐MSCs‐Exos) were prepared and combined with MJ or GA. In HepG2 cells and rat HCC organoids, exosome‐combined treatment significantly reduced IC50, increased apoptosis and necrosis, and inhibited ATP production. Transcriptomic analysis and qPCR revealed regulation of the IL‐17/MAPK/p53 axis, with downregulation of MEK1/2, CREB1, and IL17A, and activation of TP53. These findings indicate that MJ exerts anti‐HCC efficacy through multi‐component synergy, with GA as the main material basis, and hUC‐MSCs‐Exos enhance these effects by modulating IL‐17/MAPK/p53 signaling, inhibiting tumor proliferation and energy metabolism, and promoting apoptosis.

## Graphical abstract

Human‐umbilical‐cord‐mesenchymal‐stem‐cell‐derived exosomes amplify anti‐hepatocellular‐carcinoma effects of gallic acid from Compound Muji Granules. This work identifies absorbed herbal constituents and reveals modulation of IL‐17/MAPK/p53 signaling in HCC organoid models, offering mechanistic insights for exosome‐assisted traditional‐medicine anticancer strategies.

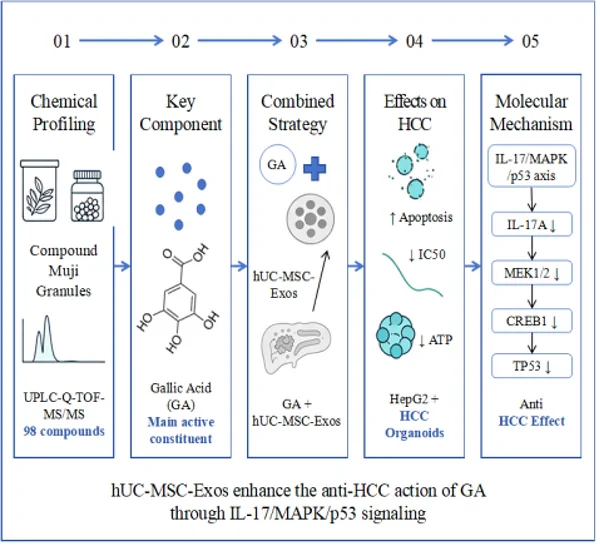

## 1 Introduction

Hepatocellular carcinoma (HCC) is one of the most prevalent malignant tumors threatening human health worldwide, with persistently high morbidity and mortality [1]. Conventional HCC treatments, including surgical resection, chemotherapy, and radiotherapy, have alleviated the disease to some extent but still present numerous limitations [2]. Surgical resection is restricted by tumor location, size, and physical conditions of patients, making it inapplicable to all cases. Chemotherapeutic agents often lead to poor patient tolerance due to severe toxic and side effects, and easily induce drug resistance in tumor cells, compromising therapeutic efficacy. Radiotherapy may damage adjacent normal tissues and trigger a series of complications [3]. Therefore, it is urgent to explore more effective and low‐toxic therapeutic strategies for HCC.

HCC is not recorded as a distinct disease name in ancient traditional Chinese medicine (TCM) classics. Based on its clinical manifestations, it can be categorized as “Zheng Jia” (abdominal mass), “Ji Ju” (accumulation), “Fei Qi”, or “Pi Qi” [4]. The TCM pathogenesis of HCC is well described as “Liver Accumulation”, mainly caused by disorders of daily living, emotional imbalance, improper diet, exogenous pathogenic factors, or erroneous treatment, leading to dysfunction of zang‐fu organs and abnormal production of essence, qi, blood, and body fluids. Over time, healthy qi is depleted, and pathogenic factors accumulate in the body, eventually resulting in tumor formation [5]. Compound Muji Granules (MJ) are derived from the Manchu folk prescription “Muji Decoction”, consisting of Coriolus versicolor extract, Sophora tonkinensis, Cuscuta chinensis, and Juglans mandshurica Maxim. It has been clinically used to inhibit the elevation of alpha‐fetoprotein (AFP). Modern studies have suggested its potential in liver protection, anti‐inflammation, and antitumor activities [6]. However, its material basis, in vivo process, action targets, and signaling pathways remain largely uncharacterized, severely restricting its precise clinical application and mechanistic translation.

Gallic acid (GA), one of the main chemical and active constituents of MJ, is a natural polyphenol compound widely distributed in plants, chemically known as 3,4,5‐trihydroxybenzoic acid with the molecular formula C_7_H_6_O_5_. It is abundant in many plants such as pomegranate [7], tea [8], and *Terminalia chebula* [9]. Previous studies have confirmed that GA possesses multiple biological activities including anti‐inflammatory, antitumor, and antioxidant effects [10, 11, 12]. In liver disease research, GA has been found to intervene in multiple pathological processes and significantly reduce liver injury markers induced by chemicals, such as alanine transaminase (ALT) and aspartate transaminase (AST) [13], indicating that it can alleviate hepatocyte damage and preserve normal liver function. Furthermore, in nonalcoholic fatty liver disease, GA administration reduces hepatic lipid accumulation, improves lipid metabolism disorders, and alleviates fatty liver symptoms [14].

Studies have demonstrated that antioxidant activity is one of the critical hepatoprotective mechanisms of GA. During normal metabolism and pathological injury, the liver generates excessive reactive oxygen species (ROS), which attack biological macromolecules in hepatocytes (e.g., lipids, proteins, and DNA) and cause oxidative stress injury. Rich in phenolic hydroxyl groups, GA exhibits strong antioxidant capacity to scavenge excessive ROS and reduce oxidative stress, thereby alleviating oxidative damage to hepatocytes. Meanwhile, GA modulates inflammation‐related signaling pathways. As inflammation plays a key role in liver disease progression, GA inhibits the activation of inflammatory factors such as nuclear factor‐κB (NF‐κB) and reduces the release of inflammatory mediators including tumor necrosis factor‐α (TNF‐α) and interleukin‐6 (IL‐6), thereby mitigating hepatic inflammation and preventing further destruction of liver tissue [15]. Collectively, GA exerts beneficial effects on liver diseases through antioxidant and anti‐inflammatory mechanisms, providing novel potential strategies and directions for drug development in liver diseases.

Recently, with advances in tumor biology, the crucial role of exosomes in tumor development has been increasingly recognized, making them attractive targets and promising drug delivery vehicles [16, 17, 18]. Exosomes are nanoscale vesicles secreted by cells that carry bioactive molecules (proteins, nucleic acids, lipids) and participate in intercellular communication and signal transduction. They play essential roles in tumor microenvironment remodeling, proliferation, migration, invasion, and immune escape of tumor cells [19]. Human umbilical cord mesenchymal stem cell‐derived exosomes (hUC‐MSCs‐Exos) have attracted extensive attention in tumor therapy due to their low immunogenicity, easy accessibility, and scalable production [20]. As natural drug delivery carriers, exosomes can improve drug stability, enhance tumor targeting and enrichment, and boost antitumor efficacy, offering a novel strategy for studying the synergistic mechanisms of TCM compounds.

In this study, UPLC‐Q‐TOF‐MS/MS was used to systematically characterize the in vitro chemical constituents, rat blood‐absorbed components, and tissue distribution of MJ, as well as to quantify the core active ingredients. Combined with hUC‐MSCs‐Exos delivery system, HepG2 cells and rat‐derived HCC organoid models were established to evaluate the anti‐HCC effects of MJ and its key component GA at cellular and organoid levels. Using transcriptome sequencing coupled with qPCR validation, we screened key targets and signaling pathways to comprehensively reveal the material basis, in vivo process, and molecular mechanism of MJ against HCC. This study aims to provide scientific evidence for its optimized clinical application and modern pharmaceutical research and development.

## 2 Methods and Materials

### 2.1 Reagents and Instruments

Compound Muji Granules were provided by Dandong Pharmaceutical Group Co., Ltd. (Dandong, China). The exosomes extraction kit for cell culture supernatant was purchased from Cyagen Biosciences (Guangzhou) Inc. (Guangzhou, China). GA, ECL luminescent solution, cell proliferation and toxicity detection kit (CCK‐8), Annexin V‐FITC/PI cell apoptosis detection kit, all of which were purchased from Dalian Meilun Biotechnology Co., Ltd (Dalian, China). β‐actin, CD63, CD81, TSG101, and ALIX monoclonal antibodies were purchased from Proteintech Group, Inc. (Wuhan, China), matrix gel was purchased from Wuhan Servicebio Technology Co., Ltd. (Wuhan, China), Tumor tissue digestion fluid, red blood cell lysis fluid, organoid cryopreservation fluid, and organoid passage digestion fluid were all purchased from BioGenous Technologies (Suzhou) Co., Ltd. (Suzhou, China).

USAUTOFLOW CO_2_ Incubator (NU‐AIRE, Germany); Water Bath (Changzhou Zhiborui Instrument Manufacturing Co., Ltd.); SUNRISE Microplate Reader (TECAN, Switzerland); Feyond—L100 Chemiluminescence Microplate Reader (Hangzhou Allsheng Instruments Co., Ltd.); ME55 Electronic analytical balance (METTLER TOLEDO Instruments Co., Ltd.); Nikon ECLIPSE Ti Research—grade Fluorescent Inverted Microscope (Nikon, Japan); Hitachi HT7700 Transmission Electron Microscope (JEOL Ltd., Japan); Nano ZS90 Nanoparticle Tracking Analyzer (Malvern, UK).

### 2.2 Cell Culture

Human umbilical cord mesenchymal stem cells (hUC‐MSCs, OriCell, Cat HUXUC‐01001) and corresponding culture medium were purchased from Cyagen Biosciences (Guangzhou) Inc. (Guangzhou, China) and grown in complete hUC‐MSCs culture medium. Human HCC HepG2 cells (RRID: CVCL_0027) were cultured in high‐glucose DMEM complete medium supplemented with 10% fetal bovine serum (FBS) and 1% penicillin‐streptomycin. All cells were incubated at 37°C in a humidified atmosphere containing 5% CO_2_.

### 2.3 Animals

Specific‐pathogen‐free (SPF) grade healthy male Sprague‐Dawley (SD) rats, weighing 200 ± 20 g, were purchased from Liaoning Changsheng Biotechnology Co., Ltd. The animal production license number is SCXK (Liao) 2020‐0001. After 1 week of acclimation, the rats were intragastrically administered with diethylnitrosamine (DEN) at a dose of 70 mg/kg once a week for 13 consecutive weeks to establish a rat model of HCC. All the animal experiments involved in this study have been approved by the Laboratory Animal Ethics Committee of Liaoning University of Traditional Chinese Medicine (Approval No.: 210000620240216).

### 2.4 Analysis on Chemical Constituents of MJ

#### 2.4.1 Analysis of In Vitro Chemical Constituents

MJ powder (5.0 g, 65 mesh) was extracted with 30 mL of 50% methanol by ultrasonication for 30 min. After cooling, the mixture was filtered, and the filtrate was passed through a 0.22 µm membrane before analysis. Data were acquired in both positive and negative ion modes using an Agilent mass spectrometer. Molecular feature extraction was performed with Agilent MassHunter Qualitative Analysis B.06.00 software. Precursor ions with mass deviation <5 ppm were selected, and potential compounds were tentatively identified by matching accurate masses and MS/MS fragmentation patterns with literature data and online databases.

#### 2.4.2 Analysis of In Vivo Constituents in Rats

MJ was dissolved in normal saline (1.35 g/mL) and administered intragastrically to rats at 20 mL/kg (equivalent to 10× the clinical human dose). Healthy SD rats were randomly divided into a blank group and an MJ group (*n* = 6 per group). The MJ group received the MJ solution once daily for 5 consecutive days, while the blank group received an equal volume of saline. After the last administration, rats were fasted for 12 h and then anesthetized with 1% pentobarbital sodium. Blood was collected from the abdominal aorta into heparinized tubes. Plasma was obtained by centrifugation, and proteins were precipitated with methanol. After drying under nitrogen, samples were redissolved in 50% methanol for analysis. Tissues (heart, liver, spleen, lung, kidney, stomach, small intestine, and thymus) were collected, homogenized in normal saline (1:5, w/v), and centrifuged. Tissue supernatants were processed using the same method as plasma samples. All samples were analyzed under the same conditions, and absorbed and tissue‐distributed components were identified by comparison with the in vitro profile [21].

#### 2.4.3 Chromatographic Conditions

An Agilent Poroshell SB‐C_18_ column (2.1 × 100 mm, 2.7 µm) was adopted. The mobile phase consisted of 0.1% formic acid aqueous solution (A)‐acetonitrile (B) for positive ion mode, and water (A)‐acetonitrile (B) for negative ion mode. Gradient elution was performed with the elution procedure shown in Table 1. The flow rate was set at 0.3 mL/min, the column temperature was maintained at 25°C, and the injection volume was 2 µL.

**TABLE 1:** Gradient elution program for qualitative analysis of chemical constituents.

Time/min	A/%	B/%	Time/min	A/%	B/%
0	98	2	22	62	38
5	98	2	30	62	38
6	90	10	32	58	42
11	90	10	40	48	52
13	82	18	45	0	100
20	82	18	52	0	100

#### 2.4.4 Mass Spectrometric Conditions

An electrospray ionization source (ESI) was used in positive and negative ion modes. The capillary voltage was set at 4000 V for positive ion mode and 3500 V for negative ion mode. Both drying gas and sheath gas were high‐purity nitrogen. The drying gas flow rate was 11 L/min at a temperature of 150°C; the nebulizer pressure was 25 psi, and the sheath gas flow rate was 10 L/min with a sheath gas temperature of 350°C. The fragmentor voltage was 125 V, and the mass scanning range was set at 50–1500 m/z. The auto MS/MS mode was adopted for data acquisition.

### 2.5 Quantitative Analysis of Main Components in MJ

#### 2.5.1 Preparation of Reference Standard Solution

Appropriate amounts of GA, matrine, chlorogenic acid, syringic acid, ellagic acid, quercitrin, luteolin and formononetin reference standards were separately weighed and dissolved with methanol. Subsequently, appropriate volumes of each standard stock solution were mixed to prepare a mixed reference standard solution with concentrations of 0.830, 0.043, 0.060, 0.040, 0.056, 0.020, 0.019, and 0.024 mg/mL, respectively.

#### 2.5.2 Preparation of Test Sample Solution

The preparation method of the test sample solution was the same as that described in 2.4.1.

#### 2.5.3 Chromatographic Conditions

An Agilent Poroshell SB‐C_18_ column (2.1 × 100 mm, 2.7 µm) was employed. The mobile phase consisted of 0.1% formic acid aqueous solution (A) and acetonitrile (B) with gradient elution procedure shown in Table 2. The flow rate was 0.4 mL/min, the column temperature was 25°C, and the injection volume was 5 µL. The detection wavelengths were set at 254, 278, and 360 nm. The wavelength was fixed at 254 nm within 0–10 min, while the maximum response values of the three wavelengths were fused for the remaining time periods. GA exhibits its maximum absorption at 278 nm; nevertheless, 254 nm with a slightly lower response was adopted during its peak elution period to achieve an overall better presentation of the full chromatogram.

**TABLE 2:** Gradient elution program for quantitative analysis of chemical constituents.

Time/min	A/%	B/%	Time/min	A/%	B/%
0	98	2	220	25	75
10	98	2	225	0	100
90	85	15	230	0	100
190	60	40			

#### 2.5.4 Quantitative Analysis of Chemical Constituents

Three batches of test sample solutions of MJ were injected and analyzed under the above chromatographic conditions. The contents of the main chemical constituents in MJ were calculated by the single‐point external standard method.

### 2.6 Preparation of Administration Solutions for MJ and Ga

#### 2.6.1 Extraction of hUC‐MSCs‐Exos

When hUC‐MSCs reached ∼80% confluence, the medium was replaced with exosome‐depleted complete medium, and cells were cultured for an additional 72 h. The culture supernatant was collected and stored at −80°C until use. Cells at passages 3–8 were used for all experiments. Exosomes were isolated from the thawed supernatant by differential centrifugation using an exosome extraction reagent, following our previously established protocol [20].

#### 2.6.2 Identification of hUC‐MSCs‐Exos

The morphology and particle size of isolated exosomes were characterized by transmission electron microscopy and nanoparticle tracking analysis, respectively. Protein concentrations of hUC‐MSCs and hUC‐MSCs‐Exos were determined using the BCA assay. Proteins were denatured, separated by SDS‐PAGE, and transferred onto PVDF membranes. Membranes were blocked with 5% skim milk for 1.5 h at room temperature, then incubated overnight at 4°C with primary antibodies against CD63 (1:10 000), CD81 (1:2000), ALIX (1:5000), and TSG101 (1:10 000). After washing, membranes were incubated with secondary antibodies for 60 min at room temperature, and signals were visualized using ECL. Marker protein expression was detected using a gel imaging system.

#### 2.6.3 Preparation of Administration Solutions

Two hundred milligrams of MJ and 200 mg of hUC‐MSCs‐Exos were added into 10 mL of PBS, and ultrasonic treatment was performed in an ice‐water bath for 10 min to assist dissolution, thus obtaining the MJ‐hUC‐MSCs‐Exos (MJE) administration solution.

A mixture of 1175 µmol (200 mg) of GA and 200 mg of hUC‐MSCs‐Exos was added into 10 mL of PBS and allowed to stand at 4°C overnight to maximize the adsorption of GA by exosomes. On the next day, ultrasonic treatment was carried out in an ice‐water bath for 10 min. Subsequently, the mixture was extruded through 400 and 200 nm polycarbonate membranes in turn using a liposome extruder, with 20 rounds of extrusion back and forth, to obtain the GA‐hUC‐MSCs‐Exos (GAE) administration solution [22].

### 2.7 Establishment of HCC Organoids

Rats with confirmed DEN‐induced HCC were euthanized, and liver tumor tissues were collected under sterile conditions. After removal of adipose and necrotic tissues, tumor specimens were rinsed twice with pre‐cooled DPBS and minced into 1–3 mm^3^ pieces. Tissue fragments were digested with 10 mL of tumor tissue digestion solution at 37°C for 30–45 min. Dissociated cells were resuspended in Matrigel, and approximately 30 µL of the cell‐Matrigel mixture was seeded into each well of a 24‐well plate. After Matrigel solidification, 500 µL of HCC organoid complete medium (Table 3) was added. The medium was refreshed every 3–4 days, and organoids were passaged approximately every 14 days. Organoids were identified by HE staining, immunohistochemistry, and immunofluorescence for AFP, CK19, and EPCAM, and compared with the original tumor tissues [23].

**TABLE 3:** Formula of complete culture medium for liver cancer organoids.

Reagent category	Component	Content	Manufacturer
Basic training medium	Advance DMEM/F12	89%	Gibco (12634010)
Serum	FBS	5%	Gibco (A5256701)
Nutritional additives	Glutamax	1%	Gibco (35050061)
Nutritional additives	B27	2%	Gibco (17504044)
Nutritional additives	N‐2 additive	1%	Gibco (17502048)
Antibiotic	Penicillin‐Streptomycin‐Glutamine	1%	Gibco (10378016)
Amino acid supplements	NEAA	1%	Gibco (11140050)
Antioxidant	N‐Acetylcysteine	200 µg/mL	Sigma‐Aldrich (A9165)
Antibiotic	Primocin	100 µg/mL	Invivo (ant‐pm)
Growth factor	EGF	0.05 µg/mL	Novoprotein (CH28)
Growth factor	HGF	0.05 µg/mL	Novoprotein (CC13)
Growth factor	FGF‐10	0.02 µg/mL	Novoprotein (CR108)
Growth factor	RSPO1	0.1 µg/mL	Novoprotein (C38P)
Growth factor	Noggin	0.1 µg/mL	Novoprotein (C028)
Growth factor	Wnt3a	0.1 µg/mL	Novoprotein (C18K)
Small molecule inhibitors	Y‐27632	3 µg/mL	Beyotime (SC0326)
Small molecule inhibitors	SB202190	3 µg/mL	Beyotime (SC0380)
Small molecule inhibitors	A83‐01	0.2 µg/mL	Beyotime (SF7917)

### 2.8 Determination of Inhibitory Activity Against Hepatoma Cells

#### 2.8.1 Screening of Administration Concentrations

HepG2 cells were seeded into 96‐well plates at 1 × 10^5^ cells/mL (100 µL/well). After adhesion, cells were treated with MJ or MJE at MJ‐equivalent concentrations of 40, 20, 10, 5, 2.5, 1.25, 0.625, and 0.3125 mg/mL, or with GA or GAE at GA‐equivalent concentrations of 400, 200, 100, 50, 25, 12.5, 6.25, and 3.125 µmol/L. Paclitaxel (PTX) was used as a positive control at 80, 40, 20, 10, 5, 2.5, 1.25, and 0.625 nmol/L. Each group included three replicate wells. After 24 h of treatment, the medium was replaced with complete medium containing 10% CCK‐8 solution, and cells were incubated for 2 h at 37°C in the dark. Absorbance was measured at 450 nm, and cell viability was calculated.

#### 2.8.2 Detection of Apoptosis and Necrosis

HepG2 cells were seeded into 6‐well plates at 1 × 10^5^ cells/mL (2 mL/well). After adhesion, cells were treated with MJ (5 mg/mL), MJE (5 mg/mL), GA (50 µmol/L), GAE (50 µmol/L), or PTX (20 nmol/L) for 24 h, with three replicates per group. Cells were washed three times with PBS, digested with EDTA‐free trypsin, and collected by centrifugation. The cell pellet was resuspended in 500 µL of 1× binding buffer, and then 25 µL of Annexin V‐FITC and 50 µL of PI were added. After gentle mixing, cells were incubated at room temperature for 10 min in the dark. Apoptosis and necrosis were analyzed by flow cytometry and fluorescence microscopy.

### 2.9 Determination of the Inhibitory Activity Against HCC Organoids

#### 2.9.1 Screening of Drug Administration Concentrations

HCC organoids were seeded into 96‐well plates (10 µL/well) and cultured for 1 week. Cells were treated with MJ or MJE at MJ‐equivalent concentrations of 100, 50, 25, 12.5, 6.25, and 3.125 mg/mL; GA or GAE at GA‐equivalent concentrations of 800, 400, 200, 100, 50, and 25 µmol/L; or PTX at 400, 200, 100, 50, 25, and 12.5 nmol/L for 72 h, with three replicates per group. After treatment, the medium was replaced with pre‐cooled complete medium containing 10% CCK‐8, and Matrigel was disrupted by pipetting. Plates were incubated at 37°C for 2 h in the dark, and absorbance was measured at 450 nm. Cell viability was calculated.

#### 2.9.2 Determination of ATP Activity in HCC Organoids

HCC organoids were seeded into 96‐well plates (10 µL/well) and cultured for 1 week. Cells were treated with MJ or MJE at 40 mg/mL (MJ‐equivalent), GA or GAE at 400 µmol/L (GA‐equivalent), or PTX at 200 nmol/L for 72 h, with three replicates per group. After discarding the medium, 20 µL of pre‐cooled lysis buffer was added, and Matrigel was disrupted by pipetting to lyse the organoids. After 5 min, 10 µL of supernatant was mixed with 100 µL of pre‐equilibrated ATP detection reagent, and luminescence (RLU) was measured using a luminometer. ATP levels were calculated.

#### 2.9.3 Detection of Apoptosis and Necrosis in HCC Organoids by Annexin V‐FITC/PI Double Staining

HCC organoids were seeded into cell culture plates and cultured for 1 week. Cells were treated with MJ or MJE at 40 mg/mL (MJ‐equivalent), GA or GAE at 400 µmol/L (GA‐equivalent), or PTX at 200 nmol/L for 72 h, with three replicates per group. After treatment, organoids were stained with Annexin V‐FITC/PI solution (20 parts 1× binding buffer, 1 part Annexin V‐FITC, 2 parts PI) for 10 min at room temperature in the dark, and apoptosis/necrosis was observed by fluorescence microscopy.

### 2.10 Transcriptome Sequencing

HCC organoids were seeded into 6‐well plates (10 drops of Matrigel per well, ∼30 µL/drop) and cultured for 1 week. Organoids were treated with MJE (40 mg/mL) or GAE (400 µmol/L) for 72 h, with a control group and three replicates per group. Total RNA was extracted using TRIzol reagent, and mRNA libraries were prepared following the standard Illumina protocol. Paired‐end sequencing was performed on an Illumina platform by Shanghai Personal Biotechnology Co., Ltd. Differential expression, enrichment, and clustering analyses were conducted using standard bioinformatics pipelines [24].

### 2.11 QPCR Analysis

Total RNA was extracted from HepG2 cells using TRIzol reagent, and 1 µg of RNA was reverse‐transcribed into cDNA using Oligo(dT) primers and MMLV reverse transcriptase. qPCR was performed with gene‐specific primers (Table 4) and SYBR Green Premix under the following conditions: 95°C for 5 min, followed by 40 cycles of 95°C for 15 s and 60°C for 30 s. Relative mRNA expression was calculated using the 2^−ΔΔCt^ method.

**TABLE 4:** Primer information.

Gene	Primer sequences (5′‐3′)	GenBank accession No.	Amplicon size (bp)
IL17A	F: TCTGAGGACAAGAACTTC R: TTGGGATTGGTATTGGTAT	NM_002190.3	77
MEK1	F: CAGTGGAGTGTTCAGTCT R: ACTTCCTCAGCATCAGAT	NM_002755.4	129
MEK2	F: CTGGACTATATTGTGAAC R: TTGATGAGGCATTTATTG	NM_030662.4	92
CREB1	F: GAGTCAGTGGATAGTGTAA R: GCATCAGAAGATAAGTCATT	NM_004379.5	98
TP53	F: AGTATTTGGATGACAGAA R: ATGTAGTTGTAGTGGATG	NM_000546.6	100
GAPDH	F: CTCTGGTAAAGTGGATATTGT R: GGTGGAATCATATTGGAACA	NM_002046.7	85

### 2.12 Statistical Analysis

All experimental data were expressed as mean ± standard deviation. Independent sample *t*‐test was adopted for comparison between groups, and a value of (*p* < 0.05) was considered statistically significant. The relevant figures were generated using KingDraw and the Weishengxin online platform (https://www.bioinformatics.com.cn/), respectively.

## 3 Analysis Results of Chemical Constituents in MJ

### 3.1 In Vitro Constituents Identification

A total of 98 compounds were identified in MJ in this study. Among them, 17 compounds belonged to flavonoids and their derivatives, including 1 flavone, 2 isoflavones, 2 flavonols, 1 dihydroflavone, 2 flavonoid glycosides, 1 isoflavonoid glycoside, 7 flavonol glycosides, and 1 flavonoid analogue (Table 5). The structural classification is shown in Figures 1, 2, 3. The major fragmentation pathways of flavonoids and their derivatives included retro‐Diels–Alder (RDA) cleavage, deglycosylation and dehydration reactions [22]. Taking Compound 82 as an example, the fragmentation pattern and identification process of flavonoids were illustrated in Figure 1B. The quasi‐molecular ion peak of Compound 82 was observed at m/z 301.0345 [M−H]^−^. After RDA cleavage, a fragment at m/z 178.9983 [M−H−RDA]^−^ was produced, which further lost CO to yield the fragment ion at m/z 151.0017 [M−H−RDA−CO]^−^. Compound 82 was preliminarily identified as quercetin.

**TABLE 5:** Information on chemical constituents of MJ.

No.	Retention time/min	Compound	Molecular formula	Ion mode	Theoretical mass	Experimental mass	Error/ppm	Fragment ions	Type
1	1.485	Choline	C_5_H_13_NO	[M+H]^+^	104.1070	104.1068	−1.92	104.1063	Alkaloid
2	1.522	Gluconic acid	C_6_H_12_O_7_	[M−H]^−^	195.0510	195.0501	−4.61	161.9662, 136.9214, 114.0246, 106.1841	Other organic acids
3	1.538	Quinic acid	C_7_H_12_O_6_	[M−H]^−^	191.0561	191.0553	−4.19	133.0291, 130.0380, 108.0203, 101.0370	Other organic acids
4	1.833	Maltose	C_12_H_22_O_11_	[M−H]^−^	341.1089	341.1073	−4.69	164.2949, 119.0310, 113.0232, 101.0239	Disaccharide
	1.844	Maltose	C_12_H_22_O_11_	[M+NH_4_]^+^	360.1500	360.1493	−1.94	201.0061, 158.9959, 127.0380, 101.0225	
5	1.912	4‐Oxoproline	C_5_H_7_NO_3_	[M−H]^−^	128.0353	128.0347	−4.69	123.0423, 118.6670, 112.9048, 109.9400	Amino acid derivative
6	2.145	Sucrose	C_12_H_22_O_11_	[M−H]^−^	341.1089	341.107	−5.57	143.0353, 119.0340, 113.0234, 101.0236	Disaccharide
7	2.156	Adenine	C_5_H_5_N_5_	[M+H]^+^	136.0618	136.0615	−2.20	136.0203, 125.2095, 119.0347, 109.0512	Purine
8	2.802	p‐Hydroxycinnamic acid	C_9_H_8_O_3_	[M+NH_4_]^+^	182.0812	182.0807	−2.75	124.0387, 120.9530, 117.0562, 111.0914	Phenolic acid
9	2.847	Pyrogallic acid	C_6_H_6_O_3_	[M−H]^−^	125.0244	125.0238	−4.80	124.0112, 116.9952, 114.9011, 103.4624	Phenol
10	2.935	1‐Ethyl‐β‐D‐galactoside	C_8_H_16_O_6_	[M+Na]^+^	231.0839	231.0833	−2.60	136.0602, 128.9417, 124.0776, 112.9690	Glycoside
11	3.292	N‐Methylcytisine	C_12_H_16_N_2_O	[M+H]^+^	205.1335	205.1332	−1.46	146.0597, 134.0565, 120.9517, 110.0960	Alkaloid
12	3.391	Gallic acid	C_7_H_6_O_5_	[M−H]^−^	169.0142	169.0134	−4.73	125.0231, 119.4685, 115.0303, 107.0213	Phenolic acid
13	3.438	Maltol	C_6_H_6_O_3_	[M−H]^−^	125.0244	125.0238	−4.80	124.0112, 123.0127, 114.9011, 103.4624	Pyrone
14	3.470	Uridine	C_9_H_12_N_2_O_6_	[M−H]^−^	243.0623	243.0612	−4.53	221.7495, 136.9345, 122.0237, 110.0243	Nucleoside
15	3.710	1‐*O*‐Monogalloylglucose	C_13_H_16_O_10_	[M−H]^−^	331.0671	331.0661	−3.02	169.0132, 151.0025, 125.0233, 107.0129	Tannin
16	3.744	Cytisine	C_11_H_14_N_2_O	[M+H]^+^	191.1179	191.1174	−2.62	148.0747, 134.0587, 120.0799, 106.0643	Alkaloid
17	3.994	Oxysophocarpine	C_15_H_22_N_2_O_2_	[M+H]^+^	263.1754	263.1748	−2.28	243.1474, 195.1484, 150.1272, 112.0751	Alkaloid
18	4.475	Adenosine	C_10_H_13_N_5_O_4_	[M+H]^+^	268.1040	268.1034	−2.24	176.9069, 136.0613, 119.0349, 109.0495	Nucleoside
19	5.138	Oxymatrine	C_15_H_24_N_2_O_2_	[M+H]^+^	265.1911	265.1907	−1.51	219.1847, 150.1271, 148.1114, 136.1110	Alkaloid
20	5.758	6‐*O*‐Monogalloylglucose	C_13_H_16_O_10_	[M−H]^−^	331.0671	331.0661	−3.02	211.0224, 169.0132, 124.0153, 107.0125	Tannin
21	5.868	Fukiic acid	C_11_H_12_O_8_	[M−H]^−^	271.0459	271.0448	−4.06	250.8875, 168.0051, 124.0154, 107.0128	Other organic acids
22	6.069	2‐*O*‐Monogalloylglucose	C_13_H_16_O_10_	[M−H]^−^	331.0671	331.0661	−3.02	211.0235, 169.0125, 124.0154, 107.0128	Tannin
23	7.020	Gentisic acid	C_7_H_6_O_4_	[M−H]^−^	153.0193	153.0187	−3.92	136.9369, 128.1630, 110.0304, 108.0203	Tannin
24	7.760	3‐*O*‐Monogalloylglucose	C_13_H_16_O_10_	[M−H]^−^	331.0671	331.0661	−3.02	211.0237, 169.0130, 124.0157, 107.0131	Tannin
25	8.063	4‐*O*‐Monogalloylglucose	C_13_H_16_O_10_	[M−H]^−^	331.0671	331.0661	−3.02	211.0236, 169.0126, 124.0156, 107.0124	Tannin
26	8.561	Protocatechuic acid	C_7_H_6_O_4_	[M−H]^−^	153.0193	153.0187	−3.92	143.9361, 136.9366, 126.3227, 108.0207	Phenolic acid
27	9.054	Matrine	C_15_H_24_N_2_O	[M+H]^+^	249.1961	249.1957	−1.61	176.1062, 150.1271, 148.1115, 110.0961	Alkaloid
28	9.343	Sophoridine	C_15_H_24_N_2_O	[M+H]^+^	249.1961	249.1957	−1.61	176.1064, 150.1275, 148.1112, 110.0961	Alkaloid
29	9.623	Sophoridinol	C_15_H_24_N_2_O	[M+H]^+^	249.1961	249.1957	−1.61	176.1065, 150.1270, 148.1114, 110.0962	Alkaloid
30	9.909	Methyl gallate	C_8_H_8_O_5_	[M−H]^−^	183.0299	183.0291	−4.37	166.8622, 146.4544, 123.0088, 108.0209	Tannin
31	10.238	Sophocarpine	C_15_H_22_N_2_O	[M+H]^+^	247.1805	247.1800	−2.02	179.1538, 150.1272, 136.1114, 108.0805	Alkaloid
32	10.496	7,11‐Dehydromatrine	C_15_H_22_N_2_O	[M+H]^+^	247.1805	247.1800	−2.02	179.1539, 150.1273, 136.1113, 108.0805	Alkaloid
33	10.971	Scopolin	C_16_H_18_O_9_	[M+H]^+^	355.1024	355.1010	−3.94	192.0383, 177.0162, 153.0189, 144.9284	Coumarin
34	11.271	1‐*O*‐Galloylglycerol	C_10_H_12_O_7_	[M−H]^−^	243.0510	243.0498	−4.94	200.5563, 169.0114, 124.0156, 107.0127	Tannin
35	12.899	Nicotinic acid	C_6_H_5_NO_2_	[M−H]^−^	122.0248	122.0245	−2.46	110.7264, 107.0938, 104.5411, 100.9708	Other organic acids
36	13.655	(3R,4S)‐4,6,8‐trihydroxy‐7‐methoxy‐3‐methyl‐3,4‐dihydroisochromen‐1‐one	C_11_H_12_O_6_	[M−H]^−^	239.0561	239.0556	−2.09	156.0463, 132.9021, 125.0225, 108.0433	Phenol
37	15.788	1‐*O*‐Syringoylglucose or its isomer	C_15_H_20_O_10_	[M−H]^−^	359.0984	359.0972	−3.34	181.0126, 152.0453, 137.0232, 121.0286	Phenolic glycoside
38	15.929	Vanillic acid	C_8_H_8_O_4_	[M−H]^−^	167.0350	167.0344	−3.59	124.0170, 112.3963, 109.0239, 108.0202	Phenolic acid
39	16.032	Lamprolobine	C_15_H_24_N_2_O_2_	[M+H]^+^	265.1911	265.1907	−1.51	150.1272, 138.1272, 124.1119, 110.0962	Alkaloid
40	16.232	Neochlorogenic acid	C_16_H_18_O_9_	[M−H]^−^	353.0878	353.0867	−3.12	191.0547, 173.0444, 161.0229, 127.0392	Other phenylpropanoids
41	16.240	2,6‐Digalloylglucose	C_20_H_20_O_14_	[M−H]^−^	483.0780	483.0758	−4.55	271.0450, 211.0225, 169.0132, 125.0230	Tannin
42	16.343	14α‐Hydroxymatrine	C_15_H_24_N_2_O_2_	[M+H]^+^	265.1911	265.1907	−1.51	152.1426, 150.1271, 138.1271, 124.1117	Alkaloid
43	16.547	Sophoranol	C_15_H_24_N_2_O_2_	[M+H]^+^	265.1911	265.1907	−1.51	152.1427, 150.1272, 138.1266, 124.1123	Alkaloid
44	16.575	Chlorogenic acid	C_16_H_18_O_9_	[M−H]^−^	353.0878	353.0867	−3.12	191.0550, 173.0439, 135.0438, 111.0442	Other phenylpropanoids
45	16.637	1‐*O*‐Syringoylglucose or its isomer	C_15_H_20_O_10_	[M−H]^−^	359.0984	359.0972	−3.34	181.0127, 152.0470, 137.0233, 121.0285	Phenolic glycoside
46	16.642	1,6‐Digalloylglucose	C_20_H_20_O_14_	[M−H]^−^	483.0780	483.0758	−4.55	271.0453, 211.0229, 169.0127, 125.0233	Tannin
47	16.739	Cryptochlorogenic acid	C_16_H_18_O_9_	[M−H]^−^	353.0878	353.0867	−3.12	191.0545, 173.0444, 135.0439, 111.0440	Other phenylpropanoids
48	16.801	Caffeic acid	C_9_H_8_O_4_	[M−H]^−^	179.0350	179.0347	−1.68	143.0193, 135.0431, 121.0283, 107.0507	Other phenylpropanoids
49	16.816	Syringic acid	C_9_H_10_O_5_	[M−H]^−^	197.0455	197.0446	−4.57	162.1956, 138.0299, 123.0073, 106.0040	Phenolic acid
50	17.011	1‐*O*‐Syringoylglucose or its isomer	C_15_H_20_O_10_	[M−H]^−^	359.0984	359.0972	−3.34	181.0128, 152.0465, 137.0233, 121.0281	Phenolic glycoside
51	17.075	3‐*O*‐Caffeoylshikimic acid	C_16_H_16_O_8_	[M−H]^−^	355.0772	355.0762	−2.82	179.0332, 161.0233, 135.0437, 133.0277	Phenolic ester
52	17.127	5‐Aminosalicylic acid	C_7_H_7_NO_3_	[M−H]^−^	152.0353	152.0348	−3.29	144.4300, 136.9348, 118.9349, 109.0233	Phenolic acid
53	17.378	4‐*O*‐Caffeoylshikimic acid	C_16_H_16_O_8_	[M−H]^−^	355.0772	355.0762	−2.82	179.0341, 161.0238, 135.0447, 133.0281	Phenolic ester
54	17.509	Octyl gallate	C_15_H_22_O_5_	[M−H]‐	281.1394	281.1384	−3.56	261.8758, 191.0333, 171.1156, 121.0650	Tannin
55	17.657	p‐Hydroxybenzaldehyde	C_7_H_6_O_2_	[M−H]^−^	121.0295	121.0289	−4.96	121.0272, 120.0172, 103.9000	Phenolic aldehyde
56	17.735	1‐*O*‐Caffeoylglycerol	C_12_H_14_O_6_	[M−H]^−^	253.0718	253.0708	−3.95	161.0224, 135.0434, 133.0278, 132.0200	Phenolic ester
57	17.922	Gentiopicroside	C_16_H_20_O_9_	[M−H]^−^	355.1035	355.1018	−4.79	175.0409, 160.0149, 148.0512, 134.0358	Iridoid glycoside
58	18.880	p‐Coumaric acid	C_9_H_8_O_3_	[M−H]^−^	163.0401	163.0395	−3.68	142.0308, 119.0493, 117.0336, 104.0248	Other phenylpropanoids
59	19.066	Suberic acid	C_8_H_14_O_4_	[M−H]^−^	173.0819	173.0813	−3.47	136.9358, 134.0363, 113.4052, 111.0800	Fatty acid
60	19.276	p‐Hydroxyacetophenone	C_8_H_8_O_2_	[M−H]^−^	135.0452	135.0449	−2.22	132.2133, 128.3627, 108.0193, 101.2985	Phenolic ketone
61	19.417	2‐Hydroxyphenylacetic acid	C_8_H_8_O_3_	[M−H]^−^	151.0401	151.0396	−3.31	121.0263, 112.2681, 108.0198, 100.4643	Phenolic acid
62	19.463	Aesculetin	C_9_H_6_O_4_	[M−H]^−^	177.0193	177.0188	−2.82	176.6763, 158.9179, 134.0303, 109.0285	Coumarin
63	19.668	5‐*O*‐Caffeoylshikimic acid	C_16_H_16_O_8_	[M−H]^−^	335.0772	335.0762	−2.82	179.0337, 161.0231, 135.0447, 133.0276	Phenolic ester
64	20.032	8‐Hydroxyoctanoic acid	C_8_H_16_O_3_	[M−H]^−^	159.1027	159.1024	−1.89	145.8877, 143.8883, 136.5243, 104.0259	Fatty acid
65	21.239	Sophoraflavonoloside	C_27_H_30_O_16_	[M−H]^−^	609.1461	609.1449	−1.97	301.0296, 270.8655, 179.9993, 102.9553	Flavonoid glycoside
66	21.589	Rutin	C_27_H_30_O_16_	[M−H]^−^	609.1461	609.1449	−1.97	561.2886, 301.0301, 271.0205, 152.0051	Flavonol glycoside
67	21.722	Naringenin‐7‐O‐β‐D‐glucopyranoside	C_21_H_22_O_10_	[M−H]^−^	433.1140	433.1121	−4.39	245.0085, 161.0233, 133.0287, 109.0290	Flavonoid glycoside
68	22.391	Hyperoside	C_21_H_20_O_12_	[M−H]^−^	463.0882	463.0868	−3.02	300.0261, 271.0229, 255.0278, 178.9978	Flavonol glycoside
69	22.991	Isoquercitrin	C_21_H_20_O_12_	[M−H]^−^	463.0882	463.0868	−3.02	300.0255, 271.0233, 255.0233, 151.0028	Flavonol glycoside
70	25.506	Azelaic acid	C_9_H_16_O_4_	[M−H]^−^	187.0976	187.0971	−2.67	147.0050, 125.7158, 117.0321, 104.0961	Fatty acid
71	26.395	Secoisolariciresinol	C_20_H_26_O_6_	[M−H]^−^	361.1657	361.1645	−3.32	145.0635, 135.0433, 121.0265, 107.0495	Lignan
72	27.998	Astragalin	C_21_H_20_O_11_	[M−H]^−^	447.0933	447.0919	−3.13	300.0258, 271.0233, 255.0282, 151.0026	Flavonol glycoside
73	28.216	Quercitrin	C_21_H_20_O_11_	[M−H]^−^	447.0933	447.0919	−3.13	300.0249, 271.0219, 178.9312, 151.0017	Flavonol glycoside
74	28.575	Catechin	C_15_H_14_O_6_	[M−H]^−^	289.0718	289.0705	−4.50	201.0544, 173.0590, 151.0400, 124.0148	Flavonoid
75	28.932	Kaempferol‐3‐O‐β‐D‐glucuronide	C_21_H_18_O_12_	[M−H]^−^	461.0725	461.0712	−2.82	328.0194, 312.9965, 297.9739, 269.9791	Flavonol glycoside
76	29.400	Afzelin	C_21_H_20_O_10_	[M−H]^−^	431.0984	431.0968	−3.71	284.0311, 255.0280, 227.0334, 163.0027	Flavonol glycoside
77	29.813	1‐Deoxyeucommiol	C_9_H_16_O_3_	[M−H]^−^	171.1027	171.1025	−1.17	151.5611, 146.8670, 136.9354, 124.6360	Iridoid
78	30.155	7,4′‐Dihydroxyflavone	C_15_H_10_O_4_	[M−H]^−^	253.0506	253.0498	−3.16	223.0388, 195.0439, 133.0292, 117.0336	Flavone
79	30.249	Sebacic acid	C_10_H_18_O_4_	[M−H]^−^	201.1132	201.1123	−4.48	174.0279, 155.6268, 116.9491, 100.1757	Fatty acid
80	30.396	Calycosin‐7‐O‐β‐D‐glucoside	C_22_H_22_O_10_	[M+COOH]^−^	491.1195	491.1177	−3.67	283.0576, 255.0645, 195.1362, 152.9365	Isoflavonoid glycoside
81	30.490	Eriodictyol	C_15_H_12_O_6_	[M−H]^−^	287.0561	287.0554	−2.44	214.9110, 175.0407, 135.0440, 108.0211	Flavonol
82	30.818	Quercetin	C_15_H_10_O_7_	[M−H]^−^	301.0354	301.0345	−2.99	178.9983, 151.0017, 121.0283, 109.0284	Flavonol
83	31.082	3,3′‐Di‐O‐methylellagic acid	C_16_H_10_O_8_	[M−H]^−^	329.0303	329.0293	−3.04	298.9811, 270.9867, 242.9918, 214.9966	Phenolic acid
84	31.464	12‐Hydroxy‐8,10‐heptadecadienoic acid	C_17_H_30_O_3_	[M+COOH]^−^	327.2177	327.2163	−4.28	270.9894, 226.9952, 201.1110, 171.1006	Fatty acid
85	31.736	Naringenin	C_15_H_12_O_5_	[M−H]^−^	271.0612	271.0603	−3.32	187.0379, 151.0018, 119.0490, 107.0128	Flavanone
86	31.985	Pinellic acid	C_18_H_34_O_5_	[M−H]^−^	329.2333	329.2319	−4.25	211.1319, 171.1009, 139.1113, 127.1114	Fatty acid
87	32.305	Rhein	C_15_H_8_O_6_	[M−H]^−^	283.0248	283.0236	−4.24	211.0390, 183.0438, 155.0497, 139.0531	Anthraquinone
88	32.731	Coumestrol	C_15_H_8_O_5_	[M−H]^−^	267.0299	267.0288	−4.12	239.0237, 195.0441, 167.0496, 119.0503	Coumarin
89	32.998	9,10,13‐Trihydroxy‐11‐octadecenoic acid	C_18_H_34_O_5_	[M−H]^−^	329.2333	329.2319	−4.25	201.1103, 171.1006, 143.1049, 127.1117	Fatty acid
90	33.060	Formononetin	C_16_H_12_O_4_	[M−H]^−^	267.0663	267.0651	−4.49	251.0335, 223.0388, 195.0441, 132.0210	Isoflavone
91	34.151	Physcion	C_16_H_12_O_5_	[M−H]^−^	283.0612	283.0604	−2.83	211.0394, 195.0435, 128.0617, 104.9669	Anthraquinone
92	36.954	Botrydial	C_17_H_26_O_5_	[M−H]^−^	309.1707	309.1695	−3.88	179.0685, 151.0740, 130.9655, 101.0596	Sesquiterpene
93	41.035	N‐Methyltyrosine	C_10_H_13_NO_3_	[M−H]^−^	194.0823	194.0815	−4.12	179.0568, 161.0855, 148.8960, 138.0176	Amino acid derivative
94	41.969	Emodin	C_15_H_10_O_5_	[M−H]^−^	269.0455	269.0449	−2.23	241.0482, 225.0530, 195.0441, 180.0565	Anthraquinone
95	42.187	Genistein	C_15_H_10_O_5_	[M−H]^−^	269.0455	269.0449	−2.23	225.0530, 210.0307, 197.0600, 105.0334	Isoflavone
96	44.912	Isocurcumenol	C_15_H_22_O_2_	[M−H]^−^	233.1547	233.1536	−4.72	225.5563, 217.1209, 182.0321, 165.8573	Sesquiterpene
97	46.260	Stearic acid	C_18_H_36_O_2_	[M−H]^−^	283.2643	283.2632	−3.88	267.0754, 195.0434, 148.0247, 119.1442	Fatty acid
98	50.559	Oleic acid	C_18_H_34_O_2_	[M−H]^−^	281.2486	281.2472	−4.98	228.5484, 171.5814, 146.4275, 122.4334	Fatty acid

**FIGURE 1:**
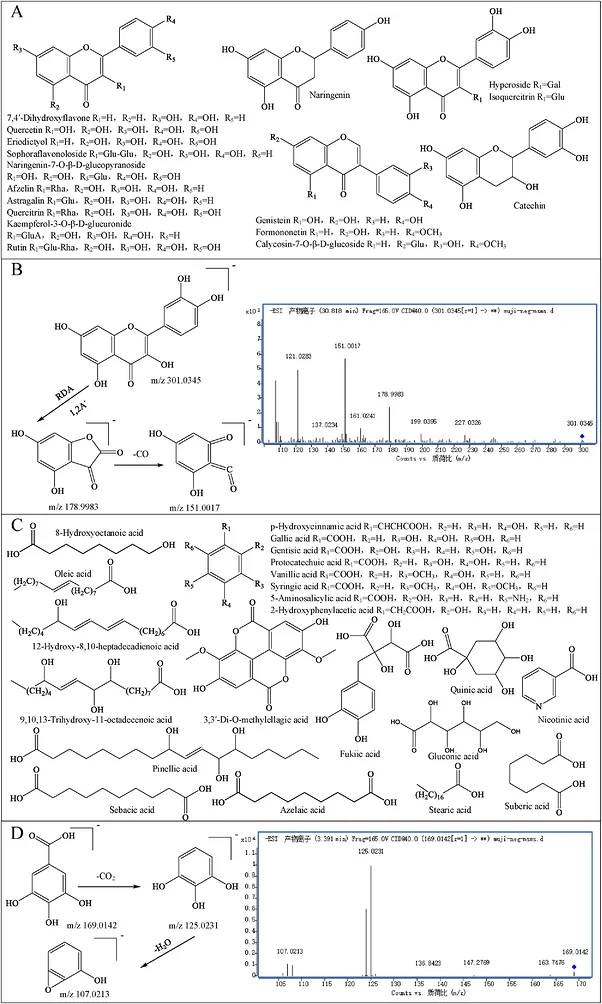
Structural classification and mass fragmentation of flavonoids and organic acids in MJ. (A) Structural types of flavonoids; (B) Fragmentation pattern and MS^2^ ions of the representative flavonoid compound (quercetin); (C) Structural types of organic acids; (D) Fragmentation pattern and MS^2^ ions of the representative organic acid compound (gallic acid).

**FIGURE 2:**
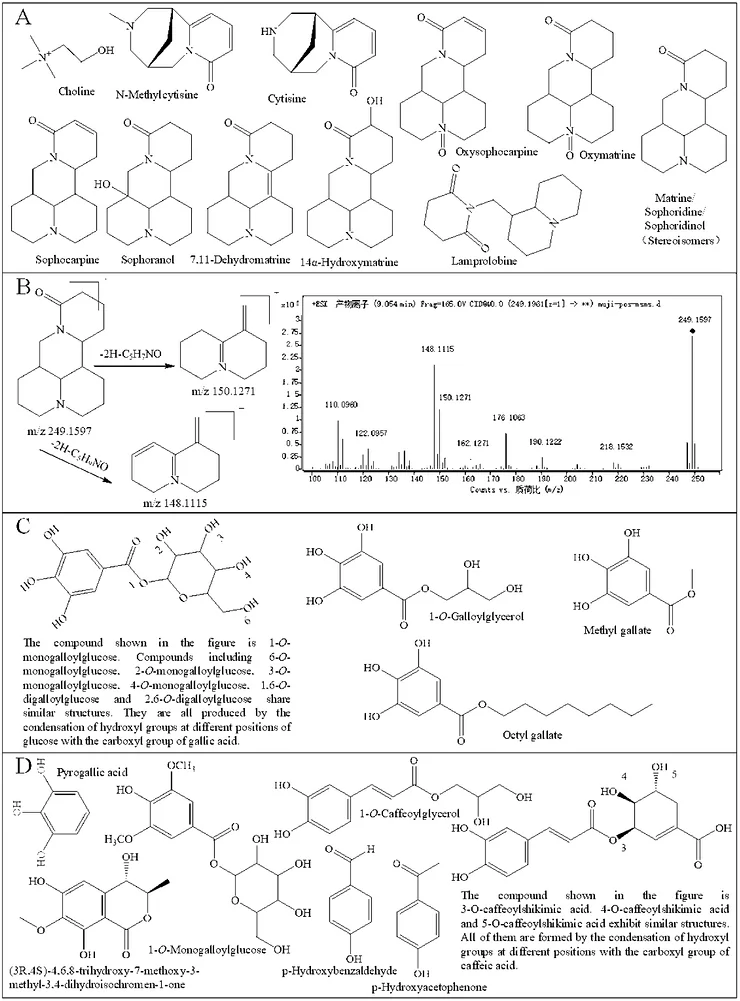
Structural classification of alkaloids, tannins and phenolic compounds in MJ. (A) Structural types of alkaloids; (B) Fragmentation pattern and MS^2^ ions of the representative alkaloid (matrine); (C) Structural types of tannins; (D) Structural types of phenolic compounds.

**FIGURE 3:**
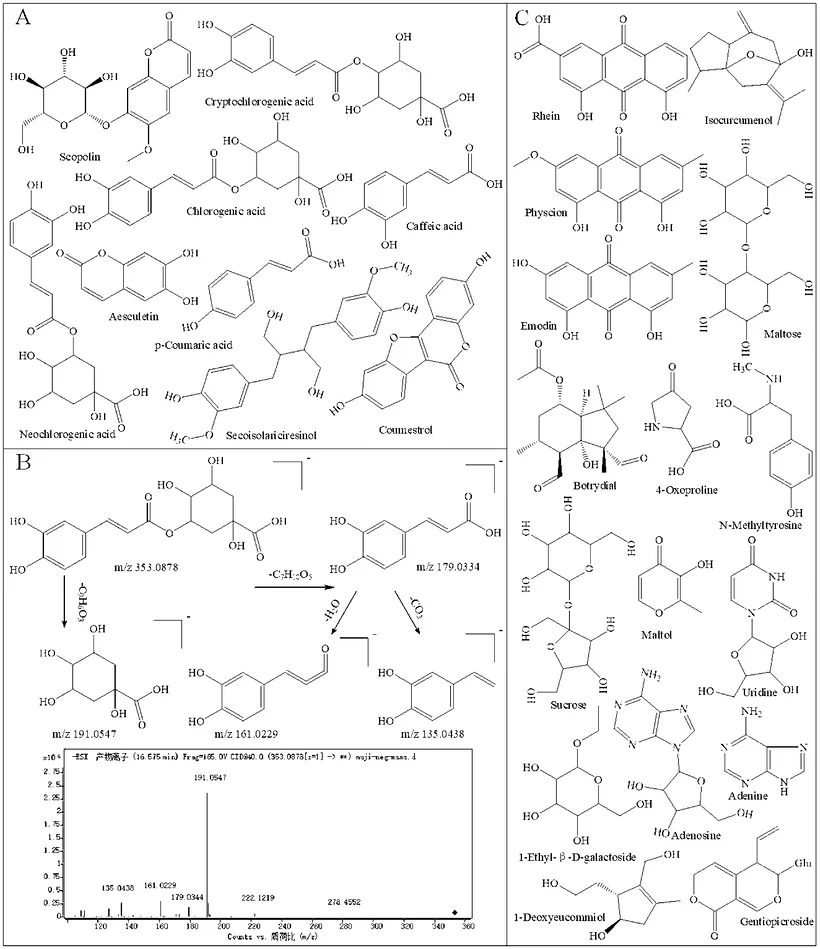
Structural characteristics of phenylpropanoids and other compounds in MJ. (A) Structure of phenylpropanoids; (B) Fragmentation pattern and MS^2^ ions of the typical phenylpropanoid (chlorogenic acid); (C) Structure of other compounds.

A total of 22 organic acids were identified, including 9 phenolic acids, 9 fatty acids, and 4 other organic acids. Their structural types are shown in Figure 1C. As illustrated in Figure 1D, Compound 12 was taken as a typical example to elaborate the identification process of phenolic acids among organic acid constituents. Other compounds such as fatty acids possess relatively simple structures, and their fragmentation patterns are not described in detail here. The quasi‐molecular ion of Compound 12 appeared at m/z 169.0142 [M−H]^−^. After decarboxylation, a fragment ion at m/z 125.0231 [M−H−COO]^−^ was generated, which further lost one molecule of H_2_O and produced the fragment at m/z 107.0216 [M−H−COO−H_2_O]^−^. Accordingly, Compound 12 identified as GA.

A total of 13 alkaloid compounds were identified, and their structural types are shown in Figure 2A. As displayed in Figure 2B, Compound 27 was selected to illustrate the fragmentation rules and identification procedure of alkaloids. The quasi‐molecular ion of Compound 27 was observed at m/z 249.1957 [M+H]^+^. Under ion source bombardment, the parent ion lost one molecule of H_2_. Single bonds between C‐5 and C‐17, C‐1 and C‐16 of the C ring, as well as C‐11 and C‐12 of the D ring were cleaved, accompanied by the removal of the D ring. Meanwhile, a double bond was formed between C‐7 and C‐11, generating the fragment ion at m/z 150.1271 [M+H−2H−C_5_H_7_NO]^+^. Alternatively, double bonds were formed between C‐7 and C‐11 as well as C‐4 and C‐5, producing the fragment ion at m/z 148.1115 [M+H−2H−C_5_H_9_NO]^+^. Thus, Compound 27 was tentatively identified as matrine.

Ten tannin compounds were characterized, and their structural types are presented in Figure 2C. A total of 11 phenols and their derivatives were also detected, including 2 phenols, 3 phenolic glycosides, 4 phenolic esters, 1 phenolone, and 1 phenolic aldehyde; their structural classification is shown in Figure 2D. Tannins and phenolic compounds possess similar frameworks to phenolic acids or contain the phenolic acid parent nucleus, and their fragmentation patterns are consistent with those of phenolic acids. Therefore, no further elaboration is given in this section.

A total of 9 phenylpropanoid compounds were identified, including 3 coumarins, 1 lignan, and 5 other phenylpropanoids. Their structural types are shown in Figure 3A. As shown in Figure 3B, Compound 44 was taken as an example to illustrate the fragmentation and identification process of phenylpropanoids. The quasi‐molecular ion of Compound 44 was at m/z 353.0878 [M−H]^−^. Fragmentation produced a quinic acid fragment at m/z 191.0547 [M−H−C_9_H_6_O_3_]^−^ and a caffeic acid fragment at m/z 179.0334 [M−H−C_7_H_10_O_5_]^−^. The caffeic acid moiety further lost H_2_O or CO_2_, yielding fragment ions at m/z 161.0229 [M−H−C_7_H_10_O_5_−H_2_O]^−^ and m/z 135.0438 [M−H−C_7_H_10_O_5_−CO_2_]^−^, respectively. Accordingly, Compound 44 was tentatively identified as chlorogenic acid.

In addition, another 16 compounds of other categories were characterized from Compound Muji Granules, including 3 anthraquinones, 1 iridoid, 1 iridoid glycoside, 2 sesquiterpenes, 2 disaccharides, 1 glycoside, 2 amino acid derivatives, 1 purine, 1 pyrone, and 2 nucleosides. Their structural types are displayed in Figure 3C. Since these compounds are not the principal constituents of MJ, their fragmentation patterns are not described in detail herein.

### 3.2 Identification of Components of MJ in Rat Plasma

Based on the chemical profiling results of Compound Muji Granules in vitro, combined with literature data and comparison of secondary mass fragment ions, a total of 25 blood‐absorbed components were identified in rat plasma, including 19 prototype components and 6 metabolites. The detailed information is listed in Table 6. The fragmentation patterns of these prototype components were consistent with those obtained from in vitro identification, which are not described further in this section.

**TABLE 6:** Information of blood‐absorbed components in rats after administration of MJ.

No.	Retention time/min	Compound	Molecular formula	Ion mode	Theoretical mass	Experimental mass	Error/ppm	Fragment ions
1	1.538	Choline^a^	C_5_H_13_NO	[M+H]^+^	104.1070	104.1068	−1.92	104.1063
2	1.681	Maltose^a^	C_12_H_22_O_11_	[M−H]^−^	341.1089	341.1077	−3.52	160.0683, 119.0338, 113.0233, 101.0236
3	1.914	p‐Coumaric acid sulfate^b^	C_9_H_8_O_6_S	[M−H]^−^	242.9969	242.9958	−4.53	136.8305, 119.0712, 111.0177, 104.0395
4	1.978	Sucrose^a^	C_12_H_22_O_11_	[M−H]^−^	341.1089	341.1077	−3.52	143.0795, 119.0337, 113.0233, 101.0237
5	3.222	N‐Methylcytisine^a^	C_12_H_16_N_2_O	[M+H]^+^	205.1335	205.1332	−1.46	146.0595, 134.0571, 120.9519, 110.0962
6	5.151	Oxymatrine^a^	C_15_H_24_N_2_O_2_	[M+H]^+^	265.1911	265.1899	−4.53	219.1844, 150.1270, 148.1112, 136.1100
7	5.926	Pyrogallol acid sulfate^b^	C_6_H_6_O_6_S	[M−H]^−^	204.9812	204.9805	−3.42	204.7380, 138.9065, 125.0231, 114.2683
8	9.006	Matrine^a^	C_15_H_24_N_2_O	[M+H]^+^	249.1961	249.1955	−2.41	176.1060, 150.1269, 148.1114, 110.0960
9	9.338	Sophoridine^a^	C_15_H_24_N_2_O	[M+H]^+^	249.1961	249.1955	−2.41	176.1061, 150.1270, 148.1113, 110.0959
10	9.609	Sophoridinol^a^	C_15_H_24_N_2_O	[M+H]^+^	249.1961	249.1957	−1.61	176.1063, 150.1274, 148.1113, 110.0960
11	10.205	Sophocarpine^a^	C_15_H_22_N_2_O	[M+H]^+^	247.1805	247.1800	−2.02	179.1539, 150.1270, 136.1116, 108.0812
12	16.816	Syringic acid^a^	C_9_H_10_O_5_	[M−H]^−^	197.0455	197.0448	−3.55	162.1272, 126.9046, 119.0492, 108.5658
13	16.975	3‐O‐Caffeoylshikimic acid^a^	C_16_H_16_O_8_	[M−H]−	355.0772	355.0762	−2.82	179.0331, 161.0238, 135.0441, 133.0276
14	17.108	5‐Aminosalicylic acid^a^	C_7_H_7_NO_3_	[M−H]^−^	152.0353	152.0348	−3.29	144.4311, 136.9352, 118.9345, 109.0233
15	17.303	Methylated gallic acid sulfate^b^	C_9_H_10_O_8_S	[M−H]^−^	277.0024	277.0011	−4.69	182.0206, 166.9973, 139.0036, 123.0079
16	17.357	Ferulic acid sulfate^b^	C_10_H_10_OS	[M−H]^−^	273.0074	273.0063	−4.03	175.0389, 157.0287, 131.0493, 108.0530
17	17.418	Gentiopicroside^a^	C_16_H_20_O_9_	[M−H]^−^	355.1035	355.1018	−4.79	193.0491, 178.0261, 161.0237, 134.0365
18	18.874	p‐Coumaric acid^a^	C_9_H_8_O_3_	[M−H]^−^	163.0401	163.0395	−3.68	154.2451, 121.0264, 119.0480, 117.0307
19	23.401	p‐Hydroxybenzoic acid^b^	C_7_H_6_O_3_	[M−H]^−^	137.0244	137.0239	−3.65	137.0219, 93.0342
20	25.334	Azelaic acid^a^	C_9_H_16_O_4_	[M−H]^−^	187.0976	187.0971	−2.67	147.0254, 125.4075, 108.0392, 100.7313
21	32.113	Rhein^a^	C_15_H_8_O_6_	[M−H]^−^	283.0248	283.0234	−4.95	211.0387, 183.0439, 155.0488, 139.2191
22	32.284	Coumestrol^a^	C_15_H_8_O_5_	[M−H]^−^	267.0299	267.0288	−4.12	239.0241, 195.0440, 167.0492, 119.0506
23	36.168	Monohydroxylated rhein^b^	C_15_H_8_O_7_	[M−H]^−^	299.0197	299.0195	−0.67	255.0302, 199.0397, 171.0449, 155.0487
24	44.796	Isocurcumenol^a^	C_15_H_22_O_2_	[M−H]^−^	233.1547	233.1536	−4.72	225.5560, 217.1215, 182.0320, 165.8577
25	50.413	Oleic acid^a^	C_18_H_34_O_2_	[M−H]^−^	281.2486	281.2472	−4.98	228.5480, 171.5820, 146.4277, 122.4330

^a^Represents prototype components.

^b^Represents metabolites.

The retention time of the blood‐absorbed component 3 was 1.914 min. The quasi‐molecular ion peak in the primary mass spectrum was observed at m/z 242.9958 [M−H]^−^, and the main secondary fragment ions were m/z 163.0387 [M−H]^−^ and m/z 119.0712 [M−H]^−^. The fragment ion at m/z 163.0387 [M−H]^−^ corresponded to the quasi‐molecular ion of p‐coumaric acid, while the fragment at m/z 119.0712 [M−H]^−^ was a characteristic fragment of p‐coumaric acid. The molecular weight of the quasi‐molecular ion at m/z 242.9958 [M−H]^−^ was 80 Da higher than that of p‐coumaric acid at m/z 163.0387 [M−H]^−^. By comparison with previous literature [25, 26], this component was speculated to be a sulfated metabolite of p‐coumaric acid, namely p‐coumaric acid sulfate.

The retention time of the blood‐absorbed component 7 was 5.926 min. Its quasi‐molecular ion peak in the primary mass spectrum was at m/z 204.9805 [M−H]^−^, and the major secondary fragment ions were m/z 204.7380 [M−H]^−^ and m/z 125.0231 [M−H]^−^. The fragment ion at m/z 125.0231 [M−H]^−^ was inferred to be generated by the loss of a sulfonic acid group, corresponding to [M−H−SO_3_]^−^. The molecular weight of the quasi‐molecular ion at m/z 204.9805 was 80 Da higher than that of pyrogallic acid at m/z 125.0238 [M−H]^−^. Combined with literature comparison [27], this component was tentatively identified as the sulfated metabolite of pyrogallic acid, namely pyrogallic acid sulfate.

The retention time of the blood‐absorbed component 15 was 17.303 min. Its quasi‐molecular ion peak in the primary mass spectrum was detected at m/z 277.0011 [M−H]^−^, with the major secondary fragment ion at m/z 182.0206. Referring to the reported literature [28], this component was tentatively identified as a methylated and sulfated derivative of GA, methylated GA sulfate.

The retention time of the blood‐absorbed component 16 was 17.357 min. Its quasi‐molecular ion peak in the primary mass spectrum was at m/z 273.0063 [M−H]^−^. The main secondary fragment ions were m/z 175.0389 [M−H]^−^, m/z 157.0287 [M−H]^−^, m/z 131.0493 [M−H]^−^, and m/z 108.0530 [M−H]^−^, which matched the characteristic fragment ions of ferulic acid or isoferulic acid. Ferulic acid and isoferulic acid were not detected in the in vitro component identification of Compound Muji Granules, whereas chlorogenic acid was successfully characterized. Ferulic acid and isoferulic acid are known as metabolic derivatives of chlorogenic acid through certain metabolic pathways. The molecular weight of the quasi‐molecular ion at m/z 273.0063 [M−H]^−^ was nearly 80 Da higher than that of ferulic acid or isoferulic acid. Combined with literature evidence [29], component 16 was tentatively identified as the sulfated product of ferulic acid or isoferulic acid derived from chlorogenic acid metabolism, namely ferulic acid sulfate.

The retention time of the blood‐absorbed component 19 was 23.401 min. Its quasi‐molecular ion peak in the full‐scan mass spectrum was observed at m/z 137.0239 [M−H]^−^, and the major secondary fragment ions were m/z 137.0219 [M−H]^−^ and m/z 93.0342 [M−H]^−^. The fragment ion at m/z 93.0342 [M−H]^−^ was attributed to the neutral loss of one carboxyl group, corresponding to the fragment of [M−H−CO_2_]^−^. By reference to the published literature [30], this component was tentatively identified as p‐hydroxybenzoic acid.

The retention time of the blood‐absorbed component 23 was 36.168 min. Its quasi‐molecular ion peak in the primary mass spectrum was at m/z 299.0195 [M−H]^−^. The major secondary fragment ions were m/z 255.0302 [M−H]^−^, m/z 227.0342 [M−H]^−^, m/z 199.0397 [M−H]^−^, m/z 183.0448 [M−H]^−^, and m/z 171.0449 [M−H]^−^. The fragment at m/z 255.0302 [M−H]^−^ was assigned to the neutral loss of one carboxyl group, corresponding to [M−H−CO_2_]^−^. The ion at m/z 227.0342 [M−H]^−^ corresponded to further loss of one carbonyl group [M−H−CO_2_−CO]^−^, while m/z 199.0397 [M−H]^−^ was derived from the continuous loss of another carbonyl group [M−H−CO_2_−2CO]^−^. The fragment at m/z 183.0448 [M−H]^−^ was attributed to the subsequent loss of one oxygen atom [M−H−CO_2_−2CO−O]^−^. By comparison with previous literature [15], its fragmentation pattern was basically consistent with that of rhein. The molecular weight of the quasi‐molecular ion at m/z 299.0203 [M−H]^−^ was 16 Da higher than that of rhein at m/z 283.0236 [M−H]^−^. Accordingly, this component was tentatively identified as monohydroxylated rhein.

### 3.3 Tissue Distribution Identification of MJ Components in Rats

Based on the in vitro chemical constituents and plasma‐absorbed components of MJ, combined with literature references and secondary fragment ion matching, a total of 23 components were identified in various rat tissues, including 20 prototype constituents and 3 metabolites. The identified compounds were listed as follows: Isocurcumenol, Calycosin‐7‐O‐β‐D‐glucoside, 4‐Oxoproline, Azelaic acid, Methyl gallate, 5‐Aminosalicylic acid, p‐Coumaric acid, GA, sebacic acid, 7,4′‐Dihydroxyflavone, Emodin, Rhein, 3,3′‐Di‐O‐methylellagic acid, p‐Hydroxycinnamic acid, Sophocarpine, 7,11‐Dehydromatrine, Matrine, Sophoridine, Sophoridinol, Oxymatrine, Ferulic acid sulfate, Methylated GA sulfate, and Pyrogallol acid sulfate (Figure 4A). In addition, eight substances including p‐Hydroxybenzaldehyde, gluconic acid, uridine, oleic acid, octadecanoic acid, Pinellic acid, choline and p‐hydroxyacetophenone were detected in both blank and drug‐administered tissue samples. These compounds were therefore regarded as endogenous substances and are not elaborated further in this section.

**FIGURE 4:**
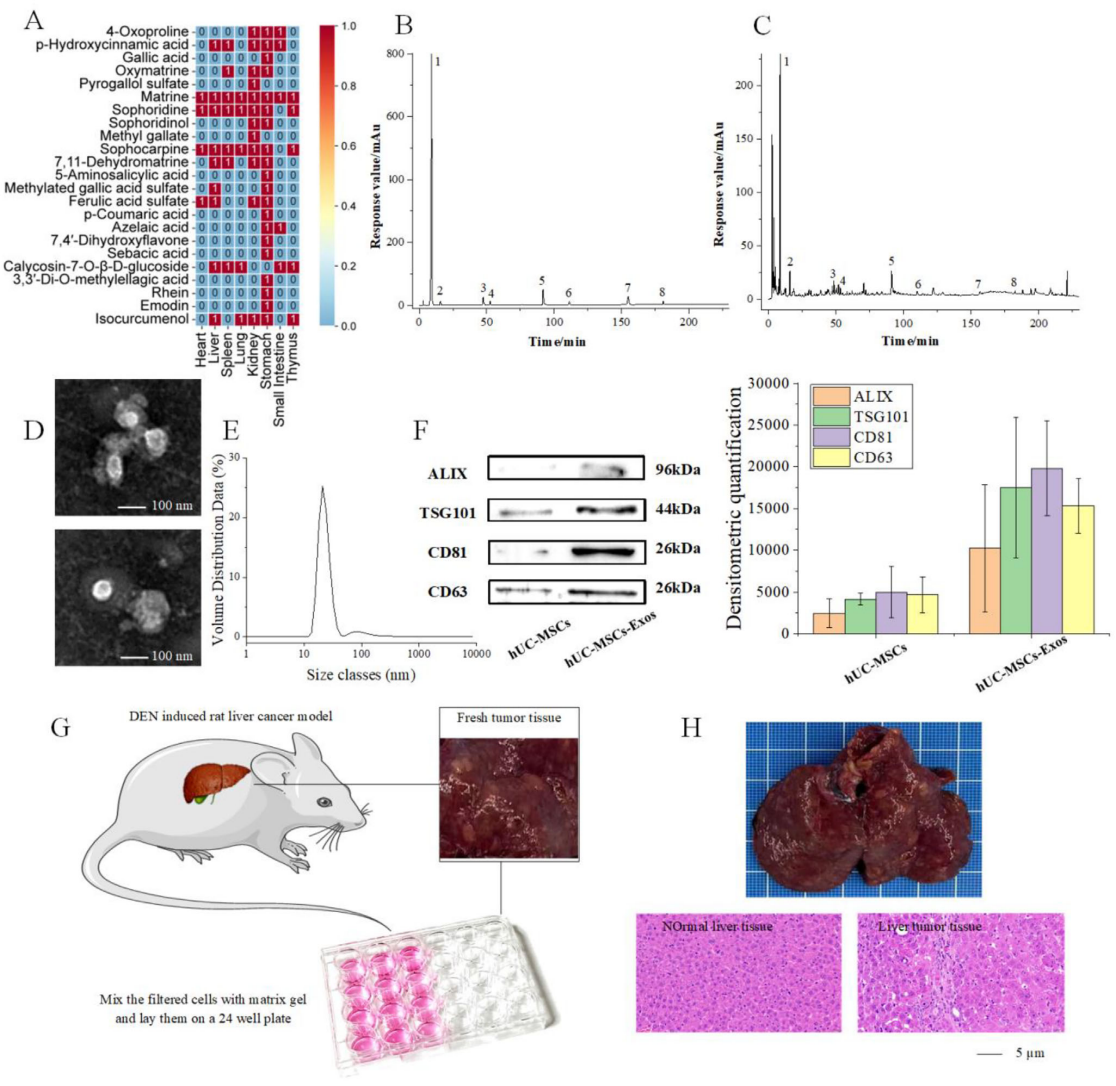
Screening of active ingredients of MJ and preliminary preparation for pharmacological verification. (A) Tissue distribution of MJ components in various rat organs (red modules and number 1 indicate positive detection in corresponding organs; blue modules and number 0 indicate undetected); (B) Chromatogram of reference substances for quantitative determination of major compounds in MJ (1: gallic acid, 2: matrine, 3: chlorogenic acid, 4: syringic acid, 5: ellagic acid, 6: quercitrin, 7: luteolin, 8: formononetin); (C) Chromatogram of sample solution for quantitative determination of major compounds in MJ (peak labels correspond to those in Figure B); (D) Transmission electron microscopic image of hUC‐MSCs‐Exos; (E) Nanoparticle size distribution analysis of hUC‐MSCs‐Exos; (F) Detection of hUC‐MSCs‐Exos marker protein expression; (G) Treatment procedure of liver cancer tissues; (H) Gross view and HE‐stained sections of liver tumor tissues.

### 3.4 Quantitative Analysis of Chemical Components

Figure 4B,C showed that the main chromatographic peaks were well separated from impurity peaks with satisfactory specificity. All analytes exhibited good linear relationships within their respective concentration ranges, as shown in Table 7. Methodological validation demonstrated that the instrumental precision, stability of the test solution within 24 h, and method repeatability all met the analytical requirements. The contents of the main chemical components in MJ were determined as follows: GA (0.8319 ± 0.0154 mg/mL), matrine (0.1116 ± 0.0055 mg/mL), chlorogenic acid (0.0071 ±  0.0003 mg/mL), syringic acid (0.0052 ± 0.0004 mg/mL), ellagic acid (0.1007 ± 0.0064 mg/mL), quercitrin (0.0014 ± 0.0001 mg/mL), luteolin (0.0009 ± 0.0001 mg/mL), and formononetin (0.0010 ± 0.0001 mg/mL).

**TABLE 7:** Regression equations and linear ranges of major compounds in MJ.

Compound	Regression equation	Correlation coefficient(r)	Linear range(mg/mL)
Gallic acid	Y = 6446.3 X + 49.07	0.9999	0.0259∼0.8300
Matrine	Y = 16179 X + 0.5572	0.9999	0.0013∼0.0430
Chlorogenic acid	Y = 10222 X + 7.5025	0.9999	0.0013∼0.0400
Syringic acid	Y = 4021.1 X + 9.2786	0.9999	0.0018∼0.0560
Ellagic acid	Y = 14294 X + 6.7562	0.9999	0.0019∼0.0600
Quercitrin	Y = 10325 X + 2.5833	0.9999	0.0013∼0.0200
Luteolin	Y = 6766.3 X + 7.5833	0.9999	0.0013∼0.0190
Formononetin	Y = 3636.2 X + 0.5833	0.9999	0.0015∼0.0240

### 3.5 Identification of hUC‐MSCs‐Exos

TEM showed that hUC‐MSCs‐Exos exhibited a typical discoid morphology (Figure 4D), consistent with exosomal ultrastructure. Nanoparticle size analysis showed an average size of 110.2 nm, mainly distributed between 20 and 200 nm (Figure 4E), within the standard exosome size range. Western blot showed high expression of the exosome markers CD63, CD81, ALIX, and TSG101 in the isolated exosomes (Figure 4F); the original Western blot images are provided in Supporting Information File 1. In summary, exosomes were successfully extracted and identified [15, 31, 32].

### 3.6 Identification of Liver Cancer Organoids

A rat liver cancer model was successfully established by DEN induction (Figure 4G,H) [33]. After successful modeling, liver tissues were isolated for the construction of liver cancer organoids. Obvious liver cancer organoids were observed on Day 3 of culture. On Day 14, the organoids grew to a size of 300–500 µm with relatively clear margins, which were distinctly distinguished from the surrounding culture environment. The construction process of liver cancer organoids is shown in Figure 5. HE staining was performed on liver cancer organoids cultured for 14 days. Microscopic observation revealed typical morphological characteristics of cancer cells in the organoids, including compact and irregular cell arrangement, enlarged and deeply stained nuclei, and an increased nucleocytoplasmic ratio, which preliminarily indicated that the constructed organoids possessed the morphological features of liver cancer cells. Immunohistochemistry and immunofluorescence results showed positive expression of CK19 in liver cancer organoids, consistent with the biological properties of liver cancer cells. As a crucial liver cancer biomarker, AFP was also positively expressed in the cytoplasm of organoid cells with strong staining intensity, further verifying the hepatic cancer characteristics of the established organoids. Meanwhile, EPCAM exhibited positive expression mainly located on the cell membrane, which was in accordance with the intrinsic biological features of liver cancer cells. In summary, the constructed organoids displayed typical characteristics of liver cancer cells in both morphological structure and biomarker expression profiles [34, 35].

**FIGURE 5:**
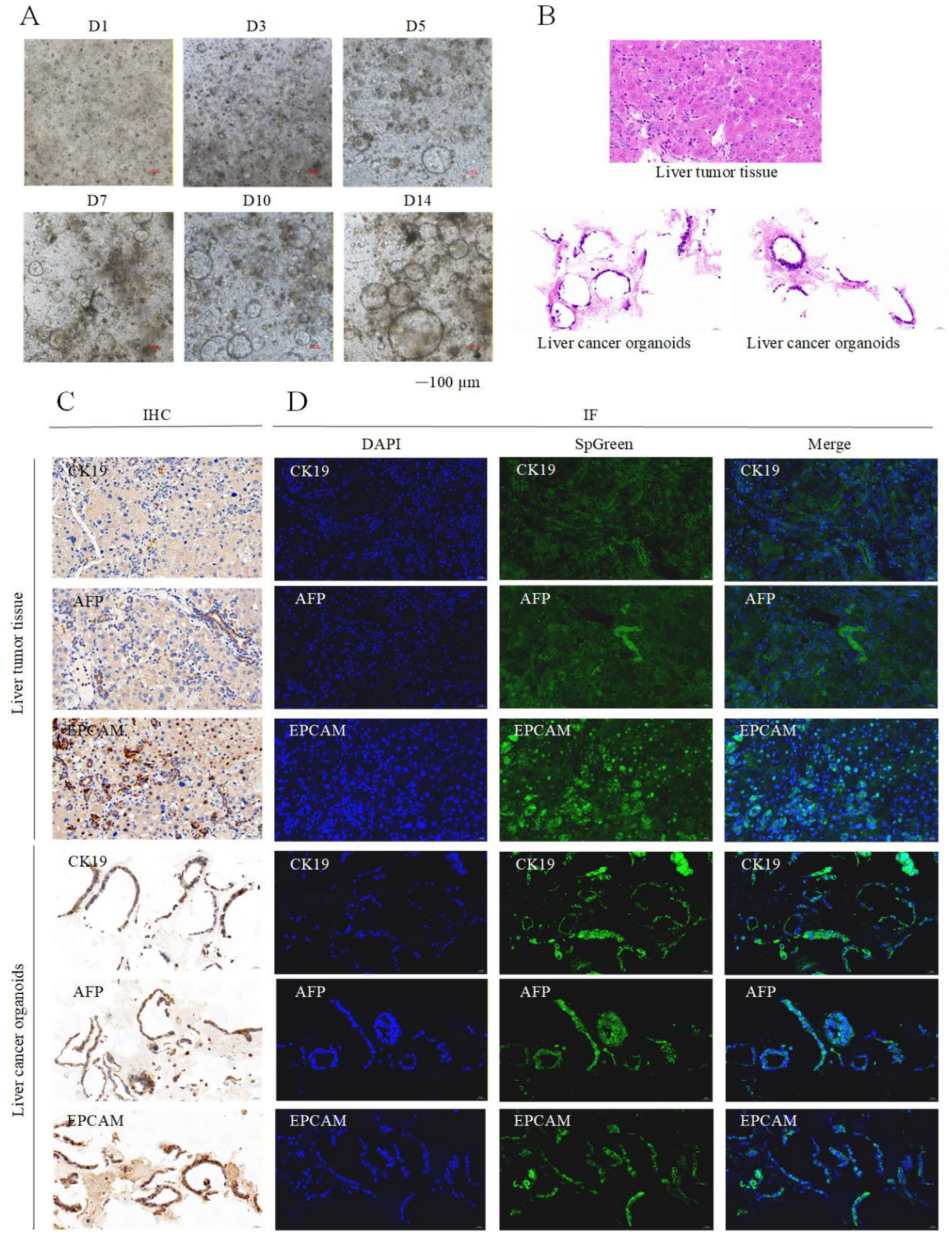
Identification of liver cancer organoids. (A) Bright‐field images of liver cancer organoids at different culture days; (B) HE‐stained sections of liver tumor tissues and liver cancer organoids; (C) Immunohistochemical staining results; (D) Immunofluorescence staining results.

### 3.7 Determination of HCC Cell Viability

After HCC cells were treated with different concentrations of drugs for 24 h, cell viability was detected by the CCK‐8 method. The results showed that the IC_50_ value of MJ against HCC cells was 6.057 ± 0.212 mg/mL, while the IC_50_ of the combined treatment with hUC‐MSCs‐Exos decreased to 4.699 ± 0.187 mg/mL. The IC_50_ of GA was 52.868 ± 0.226 µmol/L, and it decreased to 44.404 ± 0.236 µmol/L when combined with hUC‐MSCs‐Exos. The IC_50_ of PTX was 19.807 ± 0.964 nmol/L (Figure 6A–E). These findings indicated that hUC‐MSCs‐Exos could enhance the inhibitory effect of drugs on tumor cells and improve pharmacological efficacy.

**FIGURE 6:**
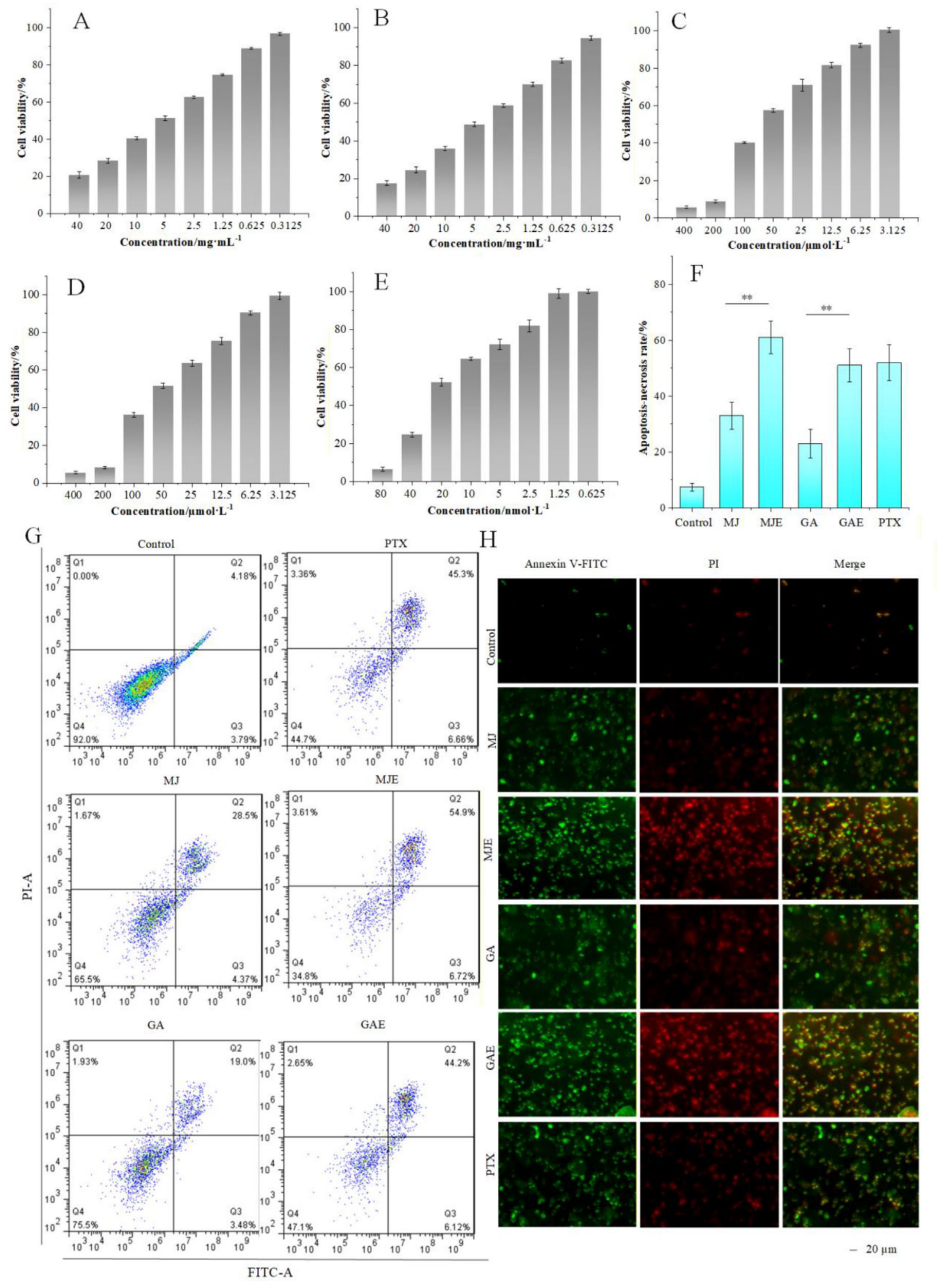
Determination of HCC cell viability. (A) Effects of different concentrations of MJ on HCC cell viability; (B) Effects of different concentrations of MJE on HCC cell viability; (C) Effects of different concentrations of GA on HCC cell viability; (D) Effects of different concentrations of GAE on HCC cell viability; (E) Effects of different concentrations of PTX on HCC cell viability; (F) Apoptosis and necrosis rates of HCC cells induced by different treatments; (G) Flow cytometry plots of HCC cell apoptosis and necrosis induced by different treatments; (H) Fluorescence images of HCC cell apoptosis and necrosis induced by different treatments.

Based on the above results, the drug concentrations for subsequent experiments were determined as follows: the concentration of MJ in both the single drug group and the combined hUC‐MSCs‐Exos group was set at 5 mg/mL; the concentration of GA in both the GA group and GA combined with hUC‐MSCs‐Exos group was set at 50 µmol/L. Consistent results were obtained from the detection of apoptosis and necrosis rates. hUC‐MSCs‐Exos significantly enhanced the inhibitory efficacy of drugs on tumor cells. The combined administration of MJ or GA with hUC‐MSCs‐Exos markedly increased the apoptosis and necrosis rates of HCC cells, as shown in Figure 6G,H.

### 3.8 Determination of HCC Organoid Viability

The CCK‐8 assay was used to evaluate the viability of HCC organoids after treatment with different concentrations of drugs for 72 h. The results showed that all drugs exerted a dose‐dependent inhibitory effect on the viability of HCC organoids. With the increase of drug concentration, the cell viability of HCC organoids gradually decreased. The IC_50_ value of MJ for inhibiting HCC organoid viability was 44.842 ± 31.10 mg/mL, while the IC_50_ decreased to 33.742 ± 25.37 mg/mL when combined with hUC‐MSCs‐Exos. The IC_50_ of GA was 427.767 ± 18.86 µmol/L, and it reduced to 266.848 ± 14.37 µmol/L in the combined hUC‐MSCs‐Exos group. The IC_50_ of PTX against HCC organoids was 205.37 ± 18.64 nmol/L (Figure 7A–E). These results indicated that the trend of drug efficacy after combined application of hUC‐MSCs‐Exos and drugs was consistent between the organoid level and the cell level. Based on comprehensive consideration, the drug concentrations for subsequent experiments were determined as follows: the concentration of MJ in both the MJ group and MJE group was set at 40 mg/mL (calculated as MJ); the concentration of GA in both the GA group and GAE group was set at 400 µmol/L (calculated as GA); and the concentration of PTX was 200 nmol/L.

**FIGURE 7:**
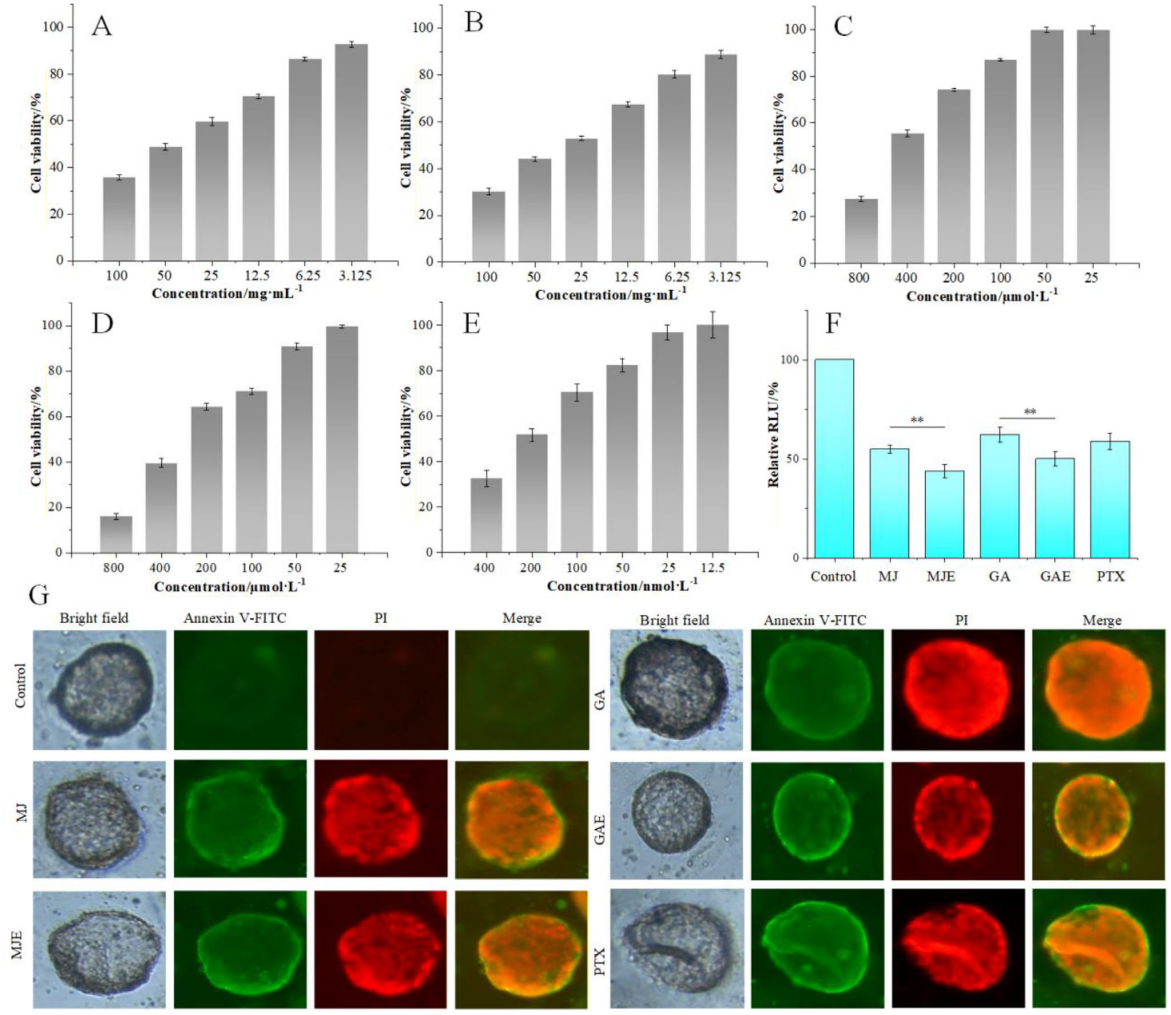
Determination of HCC organoid viability. (A) Effects of different concentrations of MJ on HCC organoid viability; (B) Effects of different concentrations of MJE on HCC organoid viability; (C) Effects of different concentrations of GA on HCC organoid viability; (D) Effects of different concentrations of GAE on HCC organoid viability; (E) Effects of different concentrations of PTX on liver cancer organoid viability; (F) Effects of different drugs on ATP activity of liver cancer organoids; (G) Fluorescence images of apoptosis and necrosis of liver cancer organoids induced by different drugs.

The inhibitory effect of drugs on the ATP content of HCC organoids was investigated. As shown in Figure 7F, the ATP content in HCC organoids gradually decreased with the increase of drug concentration, indicating that the combination of MJ or GA with hUC‐MSCs‐Exos inhibited the energy metabolism of HCC organoids, leading to a reduction in intracellular ATP production.

Finally, the optimal concentration with a suitable inhibitory effect on HCC organoid viability was selected for fluorescence photography of apoptosis and necrosis of liver cancer organoids. The results showed that obvious apoptotic and necrotic cells were observed in HCC organoids after different drug treatments, as shown in Figure 7G.

### 3.9 Transcriptome Sequencing Results

#### 3.9.1 Differentially Expressed Genes

The correlation of gene expression levels among samples is an important index to verify experimental reliability and rationality of sample selection. In this study, Pearson correlation coefficient was used to evaluate the correlation of gene expression patterns between samples; a correlation coefficient closer to 1 indicates higher similarity of expression profiles, and values between 0.8 and 1 are regarded as extremely strong correlation. Figure 8A showed that the correlation coefficients among all groups were higher than 0.9, indicating a high similarity of gene expression levels across samples. Subsequently, principal component analysis (PCA) was performed based on gene expression values. PCA can cluster samples with similar profiles together, and a shorter distance represents higher sample similarity. Figure 8B revealed good separation among groups and high aggregation within each group. Differential expression analysis was performed using Deseq2. Genes with |log2FoldChange| > 1 and *p* < 0.05 were screened as differentially expressed genes (DEGs). As shown in Figure 8C,D, a total of 1285 DEGs were identified in the MJE group, including 863 upregulated and 422 downregulated genes. In the GAE group, 1188 DEGs were obtained, among which 463 were upregulated and 725 were downregulated. These DEGs may serve as key target genes for the treatment of HCC. Bidirectional clustering analysis of the union of DEGs and samples was conducted using the R packages Pheatmap and ComplexHeatmap. Clustering was performed according to the expression patterns of individual genes across samples and the expression profiles of different genes within each sample. The Euclidean distance algorithm and complete linkage hierarchical clustering were adopted, and the clustering results are presented in Figure 8F. Based on genomic information and differential expression analysis, the R package Circlize was used to map DEGs on chromosomes and construct a genomic circle map to reflect the chromosomal distribution of DEGs (Figure 8E). Finally, protein–protein interaction (PPI) analysis was performed via the STRING database to reveal the interaction relationships among target genes, with the results shown in Figure 8G,H.

**FIGURE 8:**
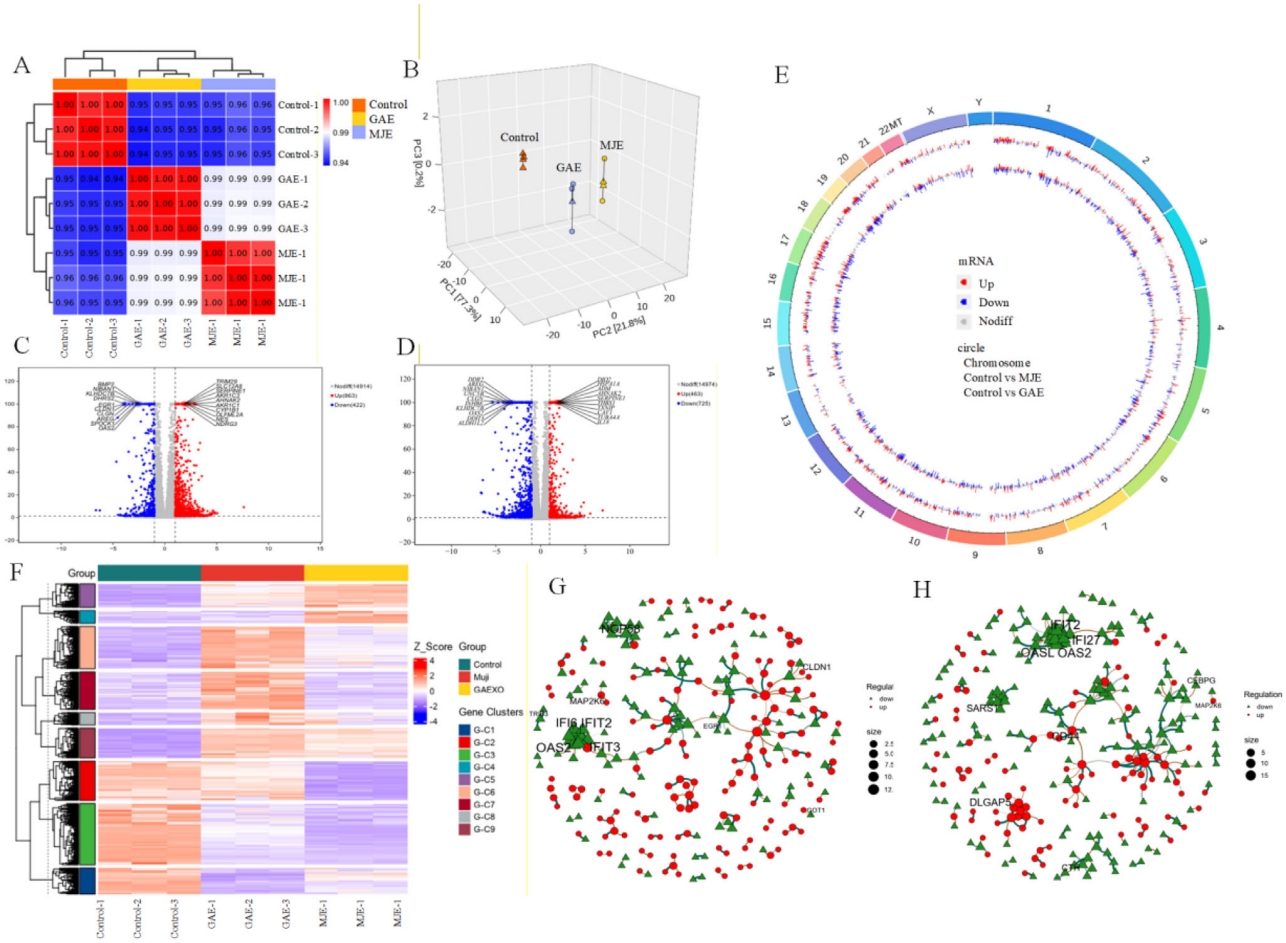
Analysis of differentially expressed genes between drug‐administered groups and control group. (A) Correlation analysis of gene expression between drug‐administered groups and control group; (B) PCA analysis of differentially expressed genes between drug‐administered groups and control group; (C) Volcano plot of differentially expressed genes between MJE group and control group; (D) Volcano plot of differentially expressed genes between GAE group and control group; (E) Genomic circle map of differentially expressed genes; (F) Cluster analysis of differentially expressed genes between drug‐administered groups and control group; (G) Protein‐protein interaction analysis of differentially expressed genes between MJE group and control group; (H) Protein‐protein interaction analysis of differentially expressed genes between GAE group and control group.

#### 3.9.2 Enrichment Analysis of Differentially Expressed Genes

Gene Ontology (GO) functional enrichment and Kyoto Encyclopedia of Genes and Genomes (KEGG) pathway enrichment analyses were performed on the DEGs. As shown in Figure 9A,B, the therapeutic effects of each drug on HCC involved multiple biological processes and signaling pathways, including the IL‐17 signaling pathway, p53 signaling pathway, and MAPK signaling pathway.

**FIGURE 9:**
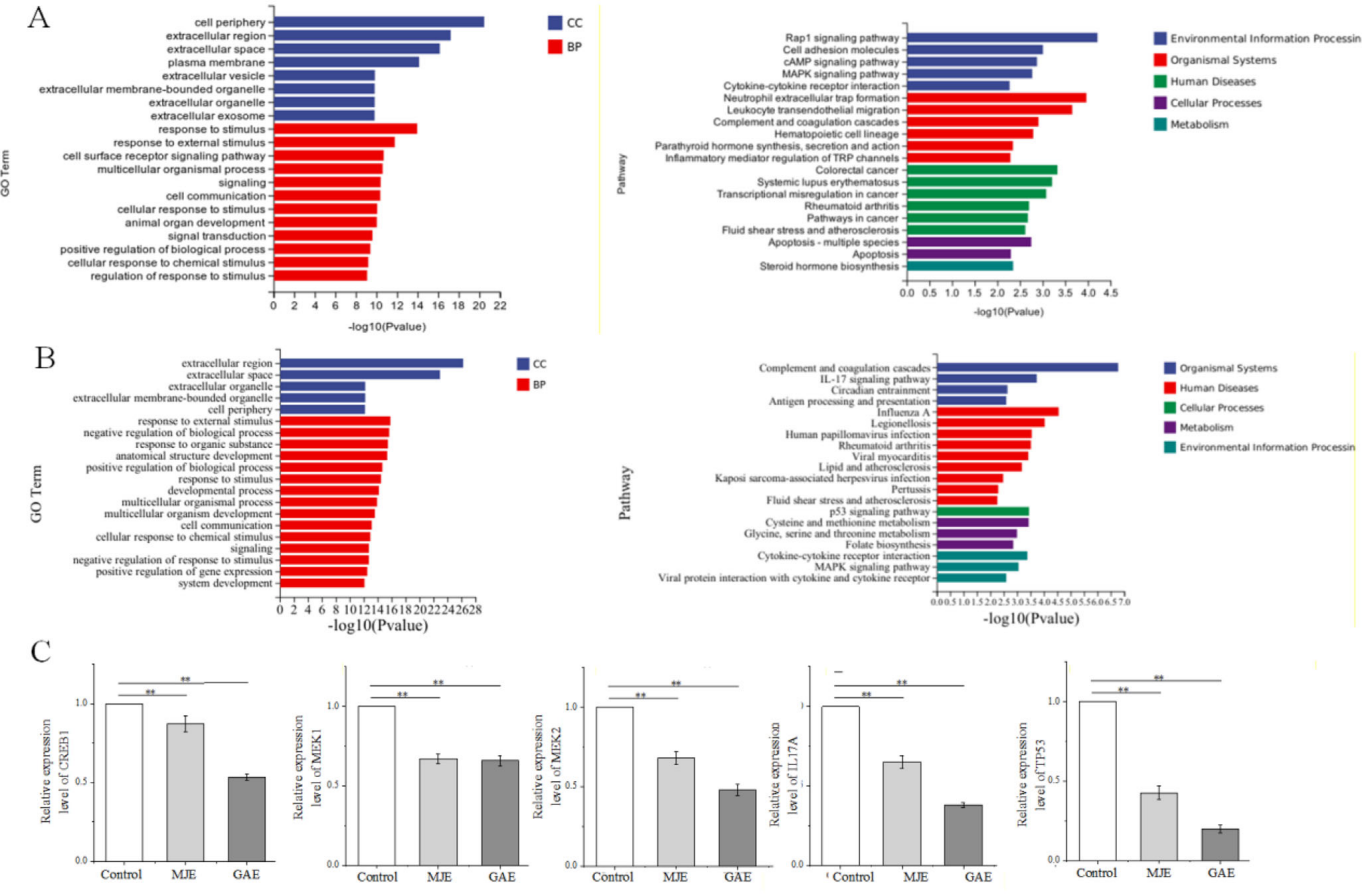
Pathway enrichment and target verification. (A) GO functional enrichment and KEGG pathway enrichment analysis of differentially expressed genes in the MJE group; (B) GO functional enrichment and KEGG pathway enrichment analysis of differentially expressed genes in the GAE group; (C) Relative mRNA expression levels of MEK1, MEK2, CREB1, IL17A, and TP53.

#### 3.9.3 QPCR Validation Results

As shown in Figure 9C, compared with the control group, the mRNA expression levels of MEK1, MEK2, CREB1, IL17A, and TP53 were significantly downregulated in the drug administration groups (*p* < 0.05).

## 4 Discussion

In the present study, a total of 98 chemical constituents were identified from MJ, covering flavonoids, organic acids, alkaloids, tannins, phenylpropanoids, and other structural types. Among them, GA, matrine, chlorogenic acid, ellagic acid, and quercitrin were selected as the core quantitative markers. In vivo results showed that 25 constituents were absorbed into blood, including 19 prototype compounds and 6 metabolites. A total of 23 components were distributed in target tissues such as liver, kidney and small intestine, suggesting that blood‐absorbed constituents constitute the material basis for its pharmacological effects. GA, matrine, oxymatrine, and rhein were identified as key effective components, among which GA exhibited the highest content in MJ.

Furthermore, a rat‐derived HCC organoid model was successfully established in this study. It was confirmed that combined administration with hUC‐MSCs‐Exos exerted a significant inhibitory effect on the viability of HCC organoids. In terms of drug screening, GA showed promising application potential, and its pharmacological mechanism was closely associated with the regulation of multiple key signaling pathways and gene expression alterations. The successful construction of HCC organoids provides an ideal in vitro model for investigating the biological characteristics of liver cancer and drug response. To a certain extent, HCC organoids can simulate the in vivo tumor microenvironment, making up for the limitations of traditional two‐dimensional cell culture and animal models.

Experimental results indicated that MJ and GA could effectively interfere with the energy metabolism of HCC organoids, thereby inhibiting tumor cell viability by disrupting cellular energy supply. Obvious apoptotic and necrotic cells were observed in drug‐treated organoids, suggesting that MJ and GA could induce programmed cell death of HCC cells. This process may involve the activation of multiple apoptotic signaling pathways, such as the mitochondrial pathway and death receptor pathway. However, the exact signal transduction mechanism remains to be further explored.

Transcriptome sequencing represents one of the key findings of this study. The screened DEGs were widely involved in biological processes including cell proliferation, differentiation, apoptosis, migration, and immune regulation. GO functional enrichment analysis indicated that these genes were mainly enriched in extracellular region, extracellular space, and response to external stimuli. KEGG pathway enrichment analysis further revealed that the IL‑17, p53, and MAPK signaling pathways were closely associated with the pharmacological effects of the drugs. The IL‑17 signaling pathway plays a crucial role in the occurrence and progression of HCC, facilitating tumor cell proliferation, angiogenesis, and the formation of an immunosuppressive microenvironment [36, 37]. It promotes tumor growth and metastasis by recruiting inflammatory cells and stimulating angiogenesis. The altered expression of genes related to the IL‑17 pathway in this study suggests that MJ and GA may exert anti‑tumor effects by targeted inhibition of the IL‑17 signaling pathway. This inhibition reduces the secretion of growth‑promoting factors, suppresses tumor angiogenesis, and modulates the function of immune cells. The MAPK signaling pathway is an essential intracellular signal transduction cascade that regulates cell proliferation, differentiation, and apoptosis. Aberrant activation of the MAPK pathway frequently occurs during tumor development. Mutations in Ras or Raf genes in various malignancies lead to persistent MAPK pathway activation, which drives cancer cell proliferation, survival, and metastasis. Moreover, the MAPK pathway is closely related to tumor drug resistance, enabling some cancer cells to evade the cytotoxicity of chemotherapeutic agents through pathway activation [38, 39, 40]. As key kinases in the MAPK cascade, MEK1, MEK2, and CREB1 exhibited markedly downregulated mRNA levels in the drug‑treated groups. These results suggest that MJ and GA may inhibit the activation of the MAPK signaling pathway, block downstream signal transduction, and consequently induce apoptosis and suppress proliferation of HCC cells.

The qPCR results further verified the reliability of transcriptome sequencing. The mRNA expression levels of MEK1, MEK2, CREB1, IL17A, and TP53 were significantly decreased in the drug administration groups. As a vital tumor suppressor gene, TP53 is involved in cell cycle regulation and DNA damage repair, and its altered expression may interfere with the above biological mechanisms [41, 42]. CREB1 participates in a variety of intracellular signal transduction and transcriptional regulation processes; downregulation of CREB1 may disrupt metabolic homeostasis and survival signaling in tumor cells [43, 44]. Collectively, the experimental findings demonstrated that combined administration with hUC‐MSCs‐Exos could target the TP53 gene as well as the IL‐17 and MAPK signaling pathways, thereby suppressing the occurrence and progression of HCC.

In addition to the direct effects on tumor cell signaling, the combined treatment may also modulate the liver–spleen axis, a systemic immunometabolic circuit that contributes to HCC progression. Beyond the direct effects on tumor cells, the synergistic activity of GA and hUC‐MSC‐Exos may also involve systemic immunometabolic regulation through the liver‐spleen axis. In HCC, the liver and spleen engage in a bidirectional circuit in which hepatocellular oxidative stress and inflammatory signals promote splenic myeloid cell priming and the subsequent recruitment of pro‐tumor immune subsets to the liver. GA, identified as the major active constituent of MJ, may attenuate this process by reducing hepatic oxidative load and metabolic disturbance, thereby limiting the upstream signals that drive splenic immune activation. In parallel, hUC‐MSC‐Exos have been reported to modulate immune polarization and suppress pro‐inflammatory cytokine release, which could further restrain the expansion of tumor‐promoting myeloid cells. Thus, the combination of GA and hUC‐MSC‐Exos may disrupt the pathological spleno‐hepatic immune trafficking that sustains tumor growth and immune evasion, providing an additional mechanistic basis for the observed in vivo synergy. This view is consistent with recent discussions of the liver‐spleen axis in HCC progression [45].

Although these systemic mechanisms provide a plausible explanation for the observed synergy, further studies are needed to directly validate the involvement of the liver–spleen axis in our model. Nevertheless, the present study still has certain limitations. Although the roles of the IL‐17/MAPK signaling pathways and several key genes have been preliminarily clarified, further in‐depth investigation is still required. The specific molecular mechanism underlying the combination of exosomes and GA remains unclear. Whether their interaction relies on physical adsorption, chemical bonding, or other forms of binding, as well as how such binding affects the biological function of exosomes and the bioavailability of GA, remains to be elucidated. In addition, the functional exploration of DEGs is not yet comprehensive. Further gene knockdown and overexpression experiments are needed to systematically dissect the specific functions and interaction relationships of each gene in the anti‑tumor process of the drugs, so as to construct a more complete molecular regulatory network.

## 5 Conclusion

In this study, a rat HCC organoid model was successfully established. The effects and underlying mechanisms of hUC‐MSCs‐Exos combined with MJ or GA on the viability of HCC organoids were systematically investigated. A series of meaningful findings were obtained, providing a novel direction for the treatment of HCC. hUC‐MSCs‐Exos served as an efficient targeted delivery carrier for the drugs, markedly enhancing their inhibitory effects on HCC organoids, and holding great potential to overcome the limitations of conventional therapeutic strategies for liver cancer. This research provides innovative insights and potential therapeutic targets for HCC treatment. Further in‐depth investigations and improvements are still required to promote the translational application of these findings into clinical practice, bringing new therapeutic prospects for liver cancer patients.

## Author Contributions

**Qiang Zhang**: writing – original draft, visualization, methodology, data curation, conceptualization. **Yige Wang**: validation, methodology. **Jinnan Huo**: validation, methodology. **Liying Han**: validation, methodology. **Ying Meng**: validation, methodology. **Yuanyuan Li**: validation. **Jiaxin Shi**: validation. **Xi Wu**: validation. **Yongrui Bao**: conceptualization, supervision. **Shuai Wang**: conceptualization, supervision. **Tianjiao Li**: funding acquisition, conceptualization. **Jiapeng Leng**: resources, conceptualization. **Xiansheng Meng**: writing – review & editing, validation, supervision, resources, conceptualization.

## Funding

This study is supported by Liaoning Provincial Natural Science Foundation Doctoral Research Startup Project (2026‐BS‐0619), 2024 Liaoning Provincial Department of Education Basic Research Project Reserve Project for Universities—Excellent Graduate Project (2024‐JYTCB‐043), Interdisciplinary Research Team for Scientific and Technological Innovation of Traditional Chinese Medicine (Under 45 Years Old), Liaoning University of Traditional Chinese Medicine (2025‐DxkjcU45‐23‐03 and 2025‐DxkjcU45‐23‐24) and General Program Project of the Joint Fund of Liaoning Provincial Department of Science and Technology (2023‐MSLH‐199).

## AI Usage Statement

The authors declare that artificial intelligence (AI) tools were used solely for language polishing to improve the clarity and readability of the manuscript. AI was not involved in figure generation, reference generation, data analysis, or any other scientific content creation. The authors take full responsibility for the scientific integrity and accuracy of the manuscript.

## Conflicts of Interest

The authors declare no conflicts of interest.

## Supporting information




**Supporting File 1**: cbdv71809‐sup‐0001‐SuppMat.docx.

## Data Availability

Data will be made available on request.
