# Supplementary material for: UPLC‐Q‐TOF‐MS/MS Profiling and Synergistic Anti‐HCC Activity of Gallic Acid From Compound Muji Granules Combined With hUC‐MSC‐Exos
Source: Chem Biodivers. 2026 Oct 11;23(10):e71809. doi: 10.1002/cbdv.71809 (PMC13655640; doi:10.1002/cbdv.71809)
Supplement: Supplementary file 1 [file CBDV-23-e71809-s001.docx]

Figure S1 Raw Western blot images for Figure 4F.

(A: TSG101; B: ALIX; C: CD81; D: CD63; 1: hUC-MSCs; 2:hUC-MSCs-Exos)
